# Identification of CD38^high^ Monocyte as a Candidate Diagnostic Biomarker and Therapeutic Target for Sepsis

**DOI:** 10.1002/advs.202500457

**Published:** 2025-03-27

**Authors:** Ning Hua, Limin Kong, Li Fan, Yanan Bai, Yujun Zhou, Yanfang Zhang, Qingwei Zhao, Xiaoyang Lu, Hongyu Yang, Hangyang Li, Peili Ding, Yuyu Nan, Qinghua Ji, Ping Yang, Lu Li, Yijing Xin, Lijuan Zhao, Wei Yang, Wenqiao Yu, Saiping Jiang

**Affiliations:** ^1^ Department of Clinical Pharmacy The First Affiliated Hospital School of Medicine Zhejiang University 79 Qingchun Road Hangzhou 310003 China; ^2^ Department of Intensive Care Unit The First Affiliated Hospital School of Medicine Zhejiang University 79 Qingchun Road Hangzhou 310003 China; ^3^ Department of Biophysics and neurology Center for Membrane Receptors and Brain Medicine the Fourth Affiliated Hospital of School of Medicine International School of Medicine International Institutes of Medicine Zhejiang University Yiwu 322000 China; ^4^ Research Center for Clinical Pharmacy College of Pharmaceutical Sciences Zhejiang University Hangzhou 310058 China; ^5^ Zhejiang Puluoting Health Technology Co Ltd Hangzhou 310000 China

**Keywords:** biomarker, CyTOF, glycolysis, scRNA‐seq, sepsis

## Abstract

Sepsis is characterized by a systemic host response to infection. Monocytes, as major mediators of acute infection, are implicated in complications among critically ill patients. Identifying key monocyte subsets and their activation states is essential for diagnosis and delineating new therapeutic targets for sepsis. Here, single cell transcriptome sequencing and mass cytometry are used to assess alterations in the composition and function of peripheral monocytes of patients with sepsis, and CD38^high^ monocytes in circulation are specifically accumulated within the first 24 h of sepsis. CD38^high^ monocytes are detectable by conventional flow cytometry to discriminate sepsis and sterile inflammation, and are associated with 28‐day mortality in bacterial sepsis. Targeting CD38 therapy markedly reduces inflammatory response in primary monocytes and in sepsis mice model. Mechanistically, CD38^high^ monocytes in sepsis exhibit hyperactivated glycolysis with activation of hypoxia‐inducible factor‐1α (HIF‐1α) due to NAD^+^ consumption. Glycolytic metabolite methylglyoxal (MGO) is found to regulate expression of CD38, establishing a CD38‐HIF‐1α/glycolysis/MGO loop that exacerbates sepsis‐induced immune dysregulation. These findings demonstrate that CD38^high^ monocytes might serve as a candidate diagnostic biomarker and therapeutic target for sepsis.

## Graphical abstract

This study uncovers that CD38^high^ monocytes discriminate sepsis and sterile inflammation and are associated with 28‐day mortality in bacterial sepsis. Targeting CD38 therapy reduces inflammatory response in monocytes and in sepsis mice model. Mechanistically, CD38‐HIF‐1α/glycolysis/MGO loop exacerbates sepsis‐induced immune dysregulation. These findings demonstrate that CD38^high^ monocytes might serve as a candidate diagnostic biomarker and therapeutic target for sepsis.

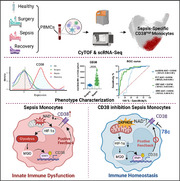

## 1 Introduction

Sepsis is a life‐threatening organ dysfunction caused by a dysregulated host response to infection, posing a significant global threat to healthy populations.^[^ ^1^ ^]^ It is a time‐sensitive condition associated with substantial mortality, morbidity, and healthcare costs.^[^ ^2^ ^]^ Early recognition and diagnosis are crucial for achieving improved outcomes, which enable clinicians to use antibiotics and targeted therapies accurately.^[^ ^3^ ^]^ Even though numerous biomarkers for sepsis have been identified, so far, no biomarker has been found reliable enough to diagnose sepsis or predict prognosis.^[^ ^4^ ^]^ The main challenges of sepsis diagnosis include syndrome complexity and heterogeneity,^[^ ^5^ ^]^ which currently depends on clinical scoring systems and the combination of multiple biomarkers.^[^ ^6^ ^]^ However, the underlying biological drivers of sepsis are limited characterized, emphasizing the need to find effective biomarkers for early sepsis diagnosis and therapy.^[^ ^7^ ^]^

Opportunities for specific signature‐led targeted and timed precision medicine approaches are limited by the lack of unbiased depictions of immune disturbances in sepsis.^[^ ^8^ ^]^ With the advancements in high‐dimensional immunoassay techniques, multi‐omics approaches have been employed to uncover comprehensive immune characteristics of sepsis,^[^ ^9^ ^]^ which is of high clinical significance to explore new immune targets. However, the existing research has yet to achieve single‐cell resolution in the exploration of cell signature and the comprehension of correlation between sepsis‐specific cell states and immune dysfunction. Myeloid cells, such as macrophages and monocytes, are associated with complications in critically ill patients and contribute to secondary fatal infections, including sepsis‐related deaths.^[^ ^10^ ^]^ Defining key cellular subsets and their activation states is a critical step in defining new diagnostic and therapeutic targets for sepsis.

To identify such sepsis‐specific cellular biomarkers, we developed single‐cell RNA sequencing (scRNA‐seq) combined with mass cytometry (CyTOF) strategy for multidimensional immunophenotyping of peripheral blood from sepsis patients in different stages at single‐cell resolution. Across distinct single‐cell profiling modalities, we identified novel CD38^high^ monocytes were specifically accumulated in sepsis patients. However, the role for CD38^high^ monocytes in sepsis has not been fully studied. Here, we illustrated that CD38^high^ monocytes were a candidate diagnostic biomarker and therapeutic factor in sepsis.

## 2 Results

### 2.1 Characteristics of the Human Participants

**Figure 1:**
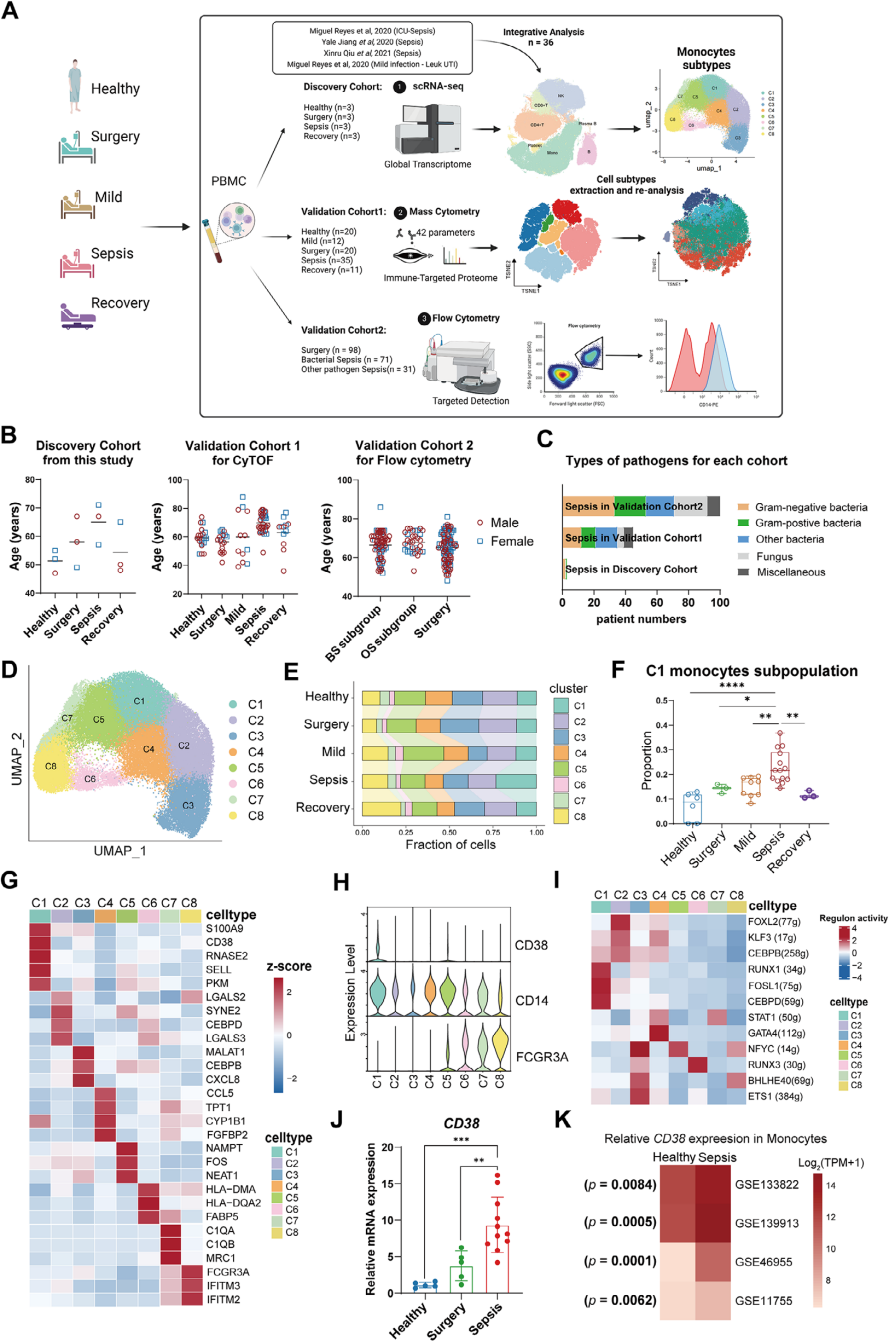
Identification of CD38^high^ monocyte as a novel signature in sepsis. A) Overview of the workflow and the experimental design for scRNA‐seq, CyTOF, and flow cytometry. The number of patients/healthy donors in each group are shown in the figure. B) Age and sex distribution of subjects and controls analyzed in this study. C) Bar plots showing sepsis patient numbers of Gram‐positive and Gram‐negative pathogens for each cohort. D) Uniform manifold approximation and projection (UMAP) visualization of subclusters shown by integrating monocytes from Healthy, Surgery, Sepsis, Recovery. E) Bar charts showing the proportions of subclusters among all monocytes in each group by scRNA‐seq. F) Violin plot showing the proportions of C1 monocytes in monocytes. Each dot represents a sample (Healthy, *n* = 6; Surgery, *n* = 3; Mild, *n* = 10; Sepsis, *n* = 14; Recovery, *n* = 3). G) Heatmap showing average expression of the highly expressed marker genes in each subcluster of monocytes. H) Violin plot showing the expression levels of *CD38*, *CD14*, and *FCGR3A* in subclusters of monocytes. Each color represents a subcluster. I) Heatmap showing the t values of AUC scores of expression regulation by transcription factors of the indicated clusters, as estimated using SCENIC. J) qPCR validation analysis of the expression level of *CD38* in CD14^+^ monocytes from different groups (Healthy, *n* = 5; Surgery, *n* = 5; Mild, *n* = 5; Sepsis, *n* = 11). K) Heatmap showing differences of mRNA expression level of *CD38* between monocytes from sepsis patients and healthy donors in published datasets. Error bars represent mean ± standard deviation (SD). **p < 0.01; ***p < 0.001. Two‐tailed unpaired Student's *t*‐test was used in K; One‐way ANOVA and Tukey post hoc tests for J.

This study comprised a scRNA‐seq discovery cohort comprised 3 patients in each group, those were patients with sepsis (Sepsis), patients undergoing cardiac surgery considered as sterile inflammation control (Surgery), patients recovered from sepsis on the seven days (Recovery), and age‐ and sex‐matched healthy donors (HC) with 14 sepsis patients and 10 mild infectious patients (Mild) sourced from public databases. CyTOF validation cohort1 included 35 Sepsis patients, 20 Surgery patients, 20 HC volunteers, 12 Mild patients, and 11 Recovery patients (Table S1, Supporting Information), and a flow cytometry (FC) validation cohort2 of 253 individuals, including 102 Sepsis patients, 53 Mild patients, and 98 Surgery patients (**Figure** **1**A). All the sepsis patients were monitored after admission, and experienced severe microbial infection and varying degrees of organ damage. Notably, the Sepsis group exhibited the most severe disease presentation, with both the acute physiology and chronic health evaluation II (APACHE II) score (*p* < 0.001) and sequential organ failure assessment (SOFA) score (*p* < 0.001) being notably higher in the sepsis group compared to the Surgery group.

### 2.2 Identification of CD38^high^ Monocyte as a Novel Signature in Sepsis Using Single‐Cell RNA Sequencing

A total of 155 608 single‐cell transcriptomes of peripheral blood mononuclear cells (PBMCs) from 12 participants (three patients with sepsis (Sepsis), three patients undergoing cardiac surgery considered as sterile inflammation control (Surgery), three patients recovered from sepsis on the seven days (Recovery), and three age‐ and sex‐matched healthy donors (HC)) (Figure 1A–C, Figure S1A,B, Supporting Information) were analyzed together with 93 363 PBMC acute sepsis datasets (all collected within 24 h of ICU admission)^[^ ^11^ ^]^ (*n* = 14), combined with datasets from urinary tract infection patients without organ dysfunction (*n* = 10) as mild infectious patients. Employing unsupervised clustering, we identified seven major cell lineages classified on the basis of canonical marker gene expression and selected monocytes with the greatest alteration in abundances and cell numbers for further analysis (Figures S1C–F and S2A–C, Supporting Information).

We subclustered monocytes into nine distinct subclusters (C1–C8) based on gene expression heterogeneity and hierarchical similarities (Figures 1D and S2D, Supporting Information), Sepsis group contained higher proportions of C1 monocytes subpopulation, and total C1 monocytes cell numbers were higher compared to HC groups, Surgery groups and Recovery groups (Figures 1E–F and S1F, Supporting Information). We found that the activation marker *CD38* was highly expressed in C1 monocytes (Figure 1G). Based on gene expression of several markers, monocytes in clusters C1, C2, C3, C4, and C5 expressed CD14; clusters C7 and C8 expressed the non‐classical monocyte marker FCGR3A (encoding CD16a) and low expression of CD14; and cluster C6 as an intermediate monocyte expressed both CD14 and FCGR3A^[^ ^12^ ^]^ (Figure 1H). We next applied single‐cell regulatory network inference and clustering (SCENIC) analysis^[^ ^13^ ^]^ and observed the unique transcriptional characteristics of CD38^high^ C1 monocytes (Figure 1I).

We re‐collected samples to determine the mRNA expression of *CD38* in CD14^+^ monocytes in each group. The qRT‐PCR results uncovered that the mRNA expression of *CD38* in the Sepsis group was significantly higher than that of patients in the Surgery group, Mild group, and HC group, thus further confirming the specificity of CD38^high^ C1 monocytes expression in patients with sepsis (Figure 1J). Meanwhile, we further analyzed independent cohorts from published bulk‐expression studies of monocytes in sepsis, and found the mRNA expression levels of *CD38* were significantly higher in patients with sepsis than in healthy controls (Figure 1K). Moreover, the expression levels of *CD38* were increased by lipopolysaccharide (LPS) stimulation of monocytes from sepsis patients compared to monocytes from healthy donors (Figure S2E, Supporting Information). The above results showed that CD38^high^ monocytes were accumulated in sepsis patients from multiple geographical locations, genetic and clinical backgrounds, which might be a novel signature in sepsis.

### 2.3 Extended Validation of CD38^high^ Monocytes Accumulated in Sepsis Using CyTOF

**Figure 2:**
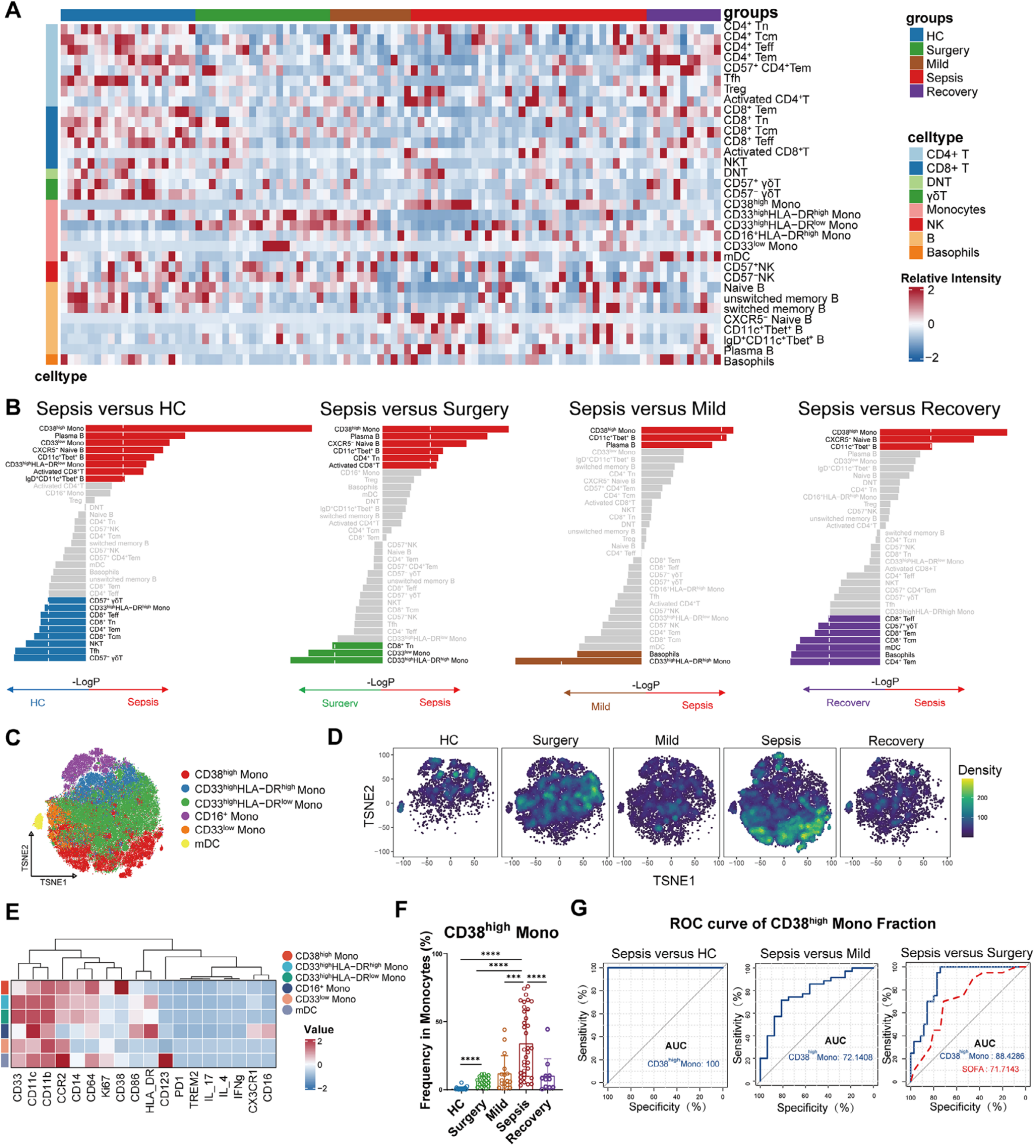
Extended validation of CD38^high^ monocytes accumulated in sepsis using CyTOF. A) Heatmap showing the relative abundance of 33 cell states identified by CyTOF, grouped by different groups (Healthy, *n* = 20; Surgery, *n* = 20; Mild, *n* = 12; Sepsis, *n* = 35; Recovery, *n* = 11). B) Comparison of cell subsets abundance between sepsis and other groups. Statistical significance was determined by a two‐sided, unpaired Wilcoxon rank‐sum test and expressed as directional −log_10_ *p* values. C) t‐SNE projection showing the components of monocyte subpopulations in CyTOF. D) Density distributions of monocytes for the HC, Surgery, Mild, Sepsis, and Recovery groups, colored by the identified monocytes clusters. E) Heatmap depicting the normalized expression of different immune marker by different monocytes subsets. F) Percentage of CD38^high^ Mono in monocytes across different groups (Healthy, *n* = 20; Surgery, *n* = 20; Mild, *n* = 12; Sepsis, *n* = 35; Recovery, *n* = 11). Statistical significance was determined by a two‐sided, unpaired Wilcoxon rank‐sum test. *p < 0.05, **p < 0.01, ***p < 0.001, ****p < 0.0001. All boxplots show median, first and third quartiles; whiskers 1.5× interquartile range. G) Receiver operating characteristic curve showing the performance of CD38^high^ Mono in distinguishing sepsis from other groups, left: Sepsis versus HC, middle: Sepsis versus Mild, right: Sepsis versus Surgery.

We performed high‐dimensional CyTOF to profile 42 leukocyte markers on peripheral blood samples to consolidate CD38^high^ monocytes at single‐cell protein level, and the information of enrolled patients was in Table S1 (Supporting Information). We performed data analysis on nearly 18 million evaluable cells, identifying 34 distinct cell subpopulations. We compared the abundance of these cellular subpopulations across different groups. Additionally, based on the infection sources of sepsis patients, we analyzed differences in immune subpopulations among patients with abdominal infections (ab_infection), pulmonary infections (p_infection), and urinary tract infections (ut_infection) (Figure S3A–E, Supporting Information). Compared to the HC group, Surgery group, Mild group, and Recovery group, respectively, sepsis patients at admission contained higher proportions of CD38^high^ Mono (**Figure** **2**A,B), which verifies the conclusion of scRNA‐seq cohort. Among patients with sepsis, higher abundance of CD38^high^ Mono was associated with the SOFA score, C‐reactive protein (CRP), and APACHE II, highlighting its potential pathophysiological significance in sepsis (Figure S3F, Supporting Information).

We further examined the characteristics of CD38^high^ Mono subsets compared with other monocyte subsets. The results of cell density dimension reduction uncovered that CD38^high^ Mono were highly expressed in Sepsis group (Figure 2C–F). Furthermore, we found CD38^high^ Mono might be used as a classifier for sepsis in the CyTOF data, with a summary area under the receiver operating characteristic curve (AUC) of 1.0 (vs HC) 0.721(vs Mild), and 0.884(vs Surgery, higher than the SOFA score) (Figure 2G).

### 2.4 CD38^high^ Monocytes Are Detectable by Conventional Flow Cytometry to Discriminate Sepsis and Non‐Infectious Systemic Inflammation

**Figure 3:**
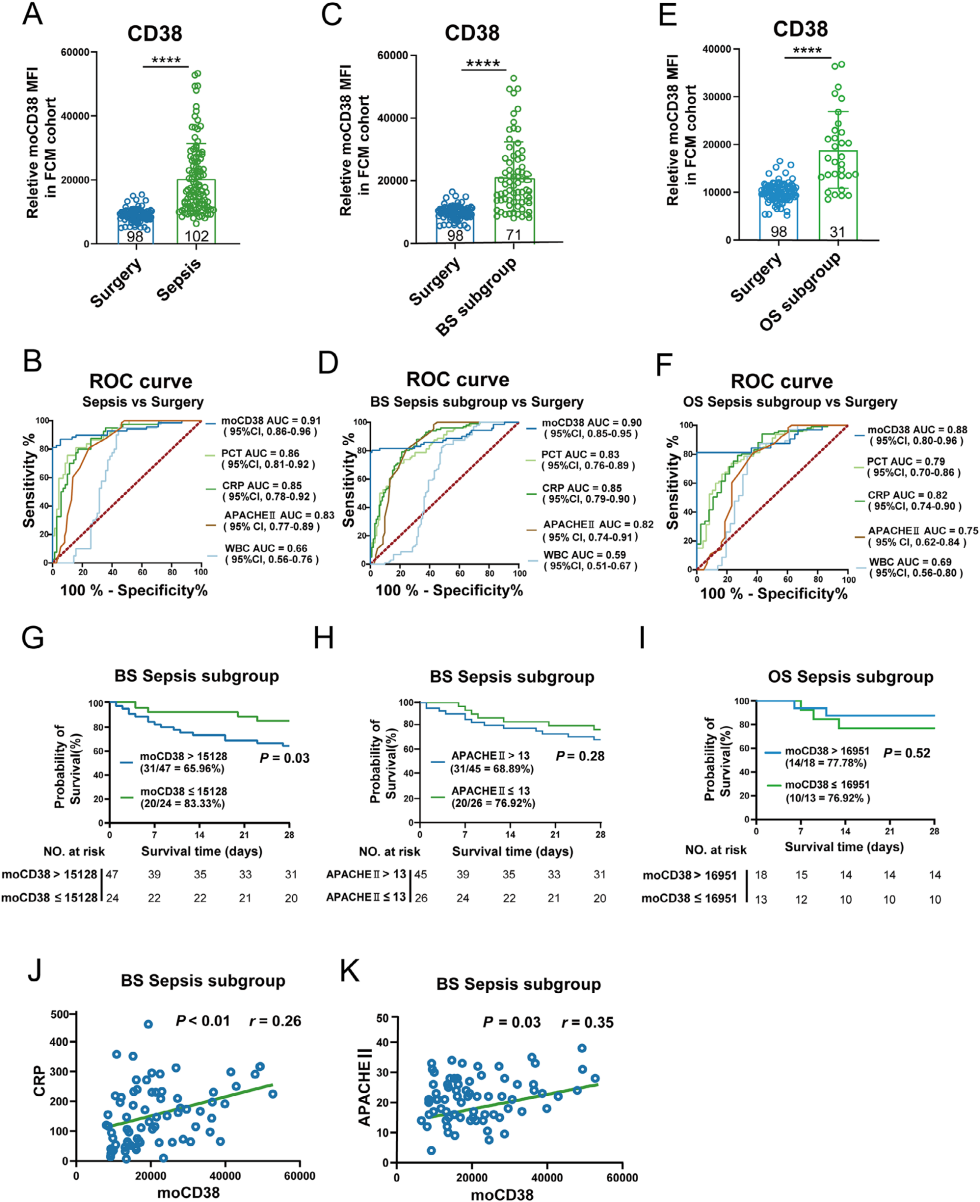
CD38^high^ monocytes are detectable by flow cytometry and discriminate sepsis patients from non‐infected patients and predict survival in bacterial sepsis patients. A) Mean fluorescence intensity of CD38 in monocytes from whole blood of patients with sepsis (*n* =  102) or patients undergoing cardiac surgery (*n* =  98). Statistical significance was determined by a Two‐tailed unpaired Student's *t*‐test. ****p < 0.0001. Error bars represent mean ± SD. B) Receiver operating characteristic curve showing the performance of the expression levels of CD38 and clinical indexes in monocytes in distinguishing sepsis from Surgery. C) Mean fluorescence intensity of CD38 in monocytes from whole blood of patients with bacterial sepsis (BS) (*n* =  71) or patients undergoing cardiac surgery (*n* = 98). Statistical significance was determined by a Two‐tailed unpaired Student's *t*‐test. ****p < 0.0001. Error bars represent mean ± SD. D) Receiver operating characteristic curve showing the performance of the expression levels of CD38 and clinical indexes in monocytes in distinguishing bacterial sepsis from Surgery. E) Mean fluorescence intensity of CD38 in monocytes from whole blood of patients with other pathogens sepsis (OS) (*n* =  31) or patients undergoing cardiac surgery (*n* = 98). Statistical significance was determined by a Two‐tailed unpaired Student's *t*‐test. ****p < 0.0001. Error bars represent mean ± SD. F) Receiver operating characteristic curve showing the performance of the expression levels of CD38 and clinical indexes in monocytes in distinguishing OS subgroup sepsis from Surgery. G) Kaplan–Meier survival curves of 71 patients with bacterial sepsis based on the expression levels of CD38 in monocytes cutoff value (15 128) on day of ICU admission. H) Kaplan–Meier survival curves of 71 patients with BS sepsis subgroup based on APACHE II cutoff value (13) on day of ICU admission. I) Kaplan‐Meier survival curves of 31 patients with OS subgroup sepsis based on the expression levels of CD38 in monocytes cutoff value (16 951) on day of ICU admission. Survival differences were evaluated via the log‐rank (Mantel–Cox) test. J) Correlation (Pearson *r*, 95% confidence interval) of the expression levels of CD38 in monocytes and CRP levels in sepsis samples (*n* =  71). K) Correlation (Pearson *r*, 95% confidence interval) of the expression levels of CD38 in monocytes and APACHEII scores in sepsis samples (*n* = 71).

To evaluate the potential diagnostic value of CD38^high^ monocyte subsets in clinical conditions, we monitored an independent validation cohort comprising 102 sepsis patients, 98 sterile inflammation patients undergoing cardiac surgery, and 53 mild infection patients (Table S2, Supporting Information). Conventional flow cytometry revealed significantly elevated CD38 expression levels of monocyte(moCD38) in sepsis patients. Furthermore, moCD38 demonstrated the highest AUC of 0.91 (95% CI: 0.86–0.96) for distinguishing the Sepsis group from the Surgery group, outperforming C‐reactive protein (CRP) concentrations, procalcitonin (PCT) concentrations, APACHE II scores, and white blood cell (WBC) counts (**Figure** **3**A,B). Our findings indicate that an MFI of CD38 ≥ 14 382 in CD14^+^ monocytes can serve as a threshold to define CD38^high^ monocytes, effectively differentiating sepsis from non‐infectious inflammation. Similar results were observed in the bacterial sepsis (BS) subgroup (moCD38 AUC = 0.90) and other pathogen‐induced sepsis (OS) subgroup (moCD38 AUC = 0.88) (Figure 3C–F). When comparing the Sepsis group, the BS subgroup sepsis, and OS subgroup sepsis with the Mild group, moCD38 levels in these sepsis groups were significantly higher than those in the Mild group (Figure S4A, Supporting Information). However, moCD38 showed AUC values of 0.82, 0.82, and 0.81 for distinguishing the Sepsis group, BS subgroup sepsis, and OS subgroup sepsis from the Mild group, respectively, with performance not surpassing clinical parameters such as CRP concentrations and PCT concentrations (Figure S4B–D, Supporting Information). These results confirm that conventional flow cytometry validates findings from single‐cell RNA sequencing (scRNA‐seq) and cytometry by time‐of‐flight (CyTOF), demonstrating the potential diagnostic value of CD38^high^ monocytes in discriminating sepsis from non‐infectious systemic inflammation.

### 2.5 CD38^high^ Monocytes Were Associated with 28‐Day Mortality in Bacterial Sepsis Patients

Kaplan–Meier analysis was performed to evaluate the association between admission level of serum CD38^high^ monocytes and clinical outcome (28‐day mortality), while there was no difference in prognosis between patients with high and low moCD38 in the Sepsis group (Figure S4E, Supporting Information). Interestingly, we found patients with higher moCD38 had poorer survival than patients with lower moCD38 (*p* = 0.03) in the BS subgroup (Figure 3G), which exhibited better predictive performance than APACHE II (Figures 3H and S4F, Supporting Information). While there was no difference in prognosis between patients with high and low moCD38 in the OS subgroup (Figure 3I). Moreover, the abundances of moCD38 were also found to positively correlate with CRP (*p* < 0.01) and APACHE II (*p* = 0.03) in the BS subgroup (Figure 3J,K). Considering the heterogeneity of sepsis, the univariate Cox regression analysis (**Table** **1**) showed age (*p* = 0.041), abdominal infection (*p* = 0.042), interleukin‐6 (IL‐6) (*p* = 0.02), SOFA score (*p* = 0.036), and moCD38 (*p* = 0.015) might be predictors of 28‐day mortality. In the subsequent multi‐variate Cox regression analysis (Table 1), the final model revealed that moCD38 (HR = 1.436, 95% CI 1.215–718, *p* = 0.019) along with SOFA score (*p* = 0.048) and IL‐6 (*p* = 0.043) were independent risk factors for 28‐day mortality.

**Table 1:** Association analysis between moCD38 levels and 28‐day mortality for bacterial sepsis patients in FC cohort.

Parameters	Univariate			Multivariate			
	HR	95% CI	*p* value	HR	95% CI		*p* value
Age	1.311	1.037–1.867	0.041	1.287	1.006	1.675	0.052
Gender	0.972	0.864–1.217	0.528				
WBC	1.281	1.032–1.483	0.362				
Infection site							
Bloodstream infection	1.915	1.182–2.473	0.611				
Abdominal infection	1.537	1.328–2.027	0.042	1.493	1.227	1.832	0.057
Respiratory tract infection	1.112	0.936–1.352	0.133				
Urinary infection	1.238	0.927–1.537	0.372				
Others infections	1.186	0.867–1.372	0.431				
IL‐6	1.638	1.352–1.839	0.02	1.722	1.328	1.934	0.043
TNF‐a	1.073	0.772–1.487	0.25				
SCR	1.047	0.936–1.384	0.173				
Bilirubin	1.281	1.002–1.792	0.087				
SOFA	1.232	1.004–1.395	0.036	1.251	1.011	1.452	0.048
APACHEII	1.033	0.981–1.127	0.413				
PCT	1.012	0.936–1.038	0.463				
CRP	1.292	1.032–1.625	0.052				
moCD38	1.379	1.181–1.642	0.015	1.436	1.215	1.718	0.019

*HR*, hazard ratio; *CI*, confidence interval; *IL‐6*, interleukin‐6; *TNF‐α*, tumor necrosis factor α; *SCR*, serum creatinine; *SOFA*, sequential organ failure assessment; *APACHE II*, acute physiology and chronic health evaluation II; *PCT*, procalcitonin; *CRP*, C‐reactive protein; *moCD38*, CD38 expression levels of monocytes.

### 2.6 Sepsis‐Induced Inflammatory Responses Depend on CD38^high^ Monocytes

**Figure 4:**
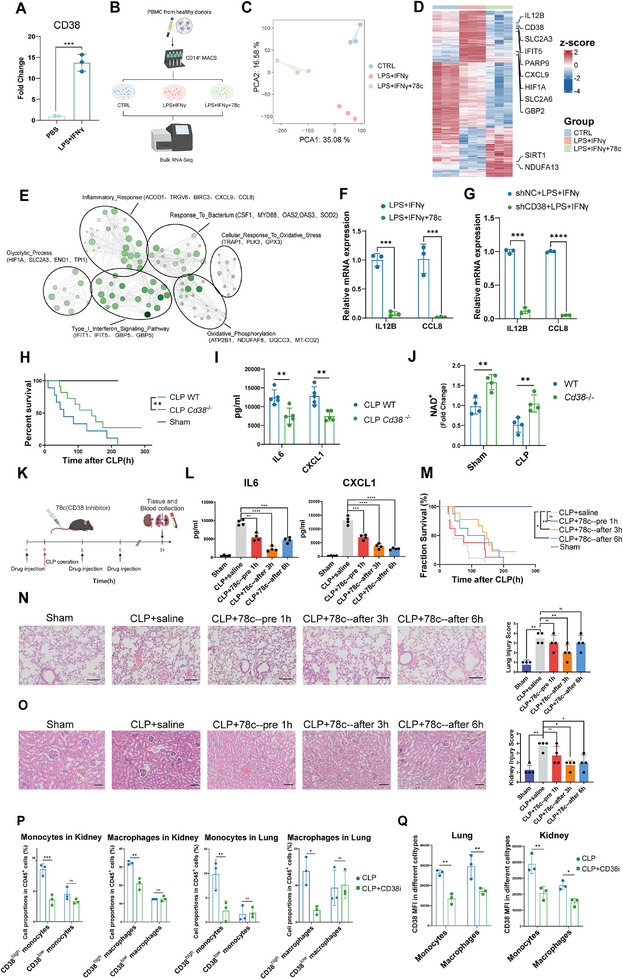
CD38 blockade and deletion alleviated sepsis inflammatory response in vitro and in vivo. A) qPCR analysis of *CD38* expression normalized to *ACTB* (*n* = 3 per group, independent experiments). B) Schema depicting the workflow for the isolation and treatment of CD14^+^ monocytes for bulk RNA‐seq (*n* = 3 per group, independent experiments). C) The principal component analysis (PCA) plot of RNA‐seq data from normal CD14^+^ monocytes (CTRL) and CD14^+^ monocytes stimulated with LPS (1 µg mL^−1^) and IFN‐γ (50 ng mL^−1^) in the presence (LPS + IFNγ + 78c) or absence (LPS + IFNγ) of CD38 inhibitor 78c (1 µm) for 6 h. D) RNA‐seq heatmap of the differentially expressed genes with highest variance (false discovery rate <0.05) among samples. E) Gene ontology network based on the differentially expressed genes between LPS + IFNγ group and LPS + IFNγ + 78c group. Each node represents a gene ontology. A selection of genes associated with each cluster of gene ontologies is displayed. F) qPCR validation analysis of the representative genes of primary monocytes normalized to *ACTB* (*n* = 3 per group, independent experiments). G) qPCR validation analysis of the representative genes of cultured monocytes THP‐1 cells with or without *CD38* knockdown normalized to *ACTB* (*n* = 3 per group, independent experiments). H) Survival rate of *Cd38* ^−/−^ mice and wild type mice subjected to CLP (*n* = 10 per group, independent experiments; log‐rank test [Mantel–Cox]). I) IL‐6 and CXCL1 levels in serum from septic mice measured by ELISA (*n* = 5 per group, independent experiments). J) Serum NAD^+^ quantification in *Cd38* ^−/−^ mice and control mice with or without sepsis modeling (*n* = 4 per group, independent experiments). K) Schematic presentation of the experimental strategy. L) IL‐6(left) and CXCL1(right) levels in serum from septic mice measured by ELISA. Intervention was administered 1 hour before modeling (CLP+78–pre 1 h) and 3 h (CLP+78–after 3 h) and 6 h (CLP+78–after 6 h) after modeling (*n* = 4 per group, independent experiments). M) Survival rates of sepsis model mice (*n* = 8 per group, independent experiments; log‐rank test [Mantel–Cox]). N) H&E staining of lung tissues in septic mice. And histological injury scores of the lungs in different groups were quantified (*n* = 4 per group, independent experiments). Each scale bar represents 200 µm. O) H&E staining of kidney tissues in septic mice. And histological injury scores of the kidneys in different groups were quantified (*n* = 4 per group, independent experiments). Each scale bar represents 200 µm. P) Flow cytometry analysis of the proportion of CD38^high^ monocytes and macrophages in kidney and lung tissues (*n* = 3 per group, independent experiments). Q) Flow cytometry analysis of the CD38 MFI of monocytes and macrophages in kidney and lung tissues (*n* = 3 per group, independent experiments). All data are representative of at least two independent experiments performed in triplicate. Error bars represent mean ± SD.*p < 0.05; **p < 0.01; ***p < 0.001; ****p < 0.0001; ns, no significant difference (*p* > 0.05). Two‐tailed unpaired Student's *t*‐test was used in A, F, G, I, J, P, Q; One‐way ANOVA and Tukey post hoc tests for L, N, O.

To explore whether CD38 drove dysfunctional activation of inflammation in monocytes of sepsis, we detected an induction of *CD38* expression in primary CD14^+^ monocytes upon LPS and IFN‐γ stimulation and performed bulk RNA‐sequencing (**Figure** **4**A,B). We found a distinct transcriptional signature of monocytes, which implied that inhibition of CD38 changed the gene expression pattern of monocytes stimulated with LPS and IFN‐γ (Figure 4C,D). Further, differentially expressed genes (DEGs) (Figure 4E and Figure S5B, Supporting Information) were found to be mainly enriched in inflammatory response (*ACOD1*, *CXCL9*), type I interferon signaling pathway (*IFIT1*, *GBP5*), glycolytic process (*HIF1A*, *SLC2A3*), cellular response to oxidative stress (*TRAP1*, *GPX3*), and oxidative phosphorylation (*NDUFAF8*, *MT‐CO2*). Moreover, *CD38* stable knockdown cell line in cultured monocytes THP‐1 cells (Figure S5D,E, Supporting Information) indicated that inhibition and knockdown of *CD38* resulted in a dramatic decrease in cytokines expression level (Figure 4F,G).

A *Cd38*‐deficient mouse model lacking exons 2–4 of the *Cd38* gene was generated using the CRISPR‐Cas9 approach to assess the impact of CD38 on sepsis. Using an in vivo cecal ligation and puncture (CLP) sepsis model, *Cd38* ablation significantly improved the survival rate of septic mice and reduced serum cytokines (Figure 4H,I). An increase in the serum NAD^+^ content was also observed in *Cd38*‐deficient mice in CLP models (Figure 4J). We next examined the phenotype of sepsis in vivo through pharmaceutical inhibition of CD38 at different time points (Figure 4K). Serum levels of interleukin‐6 (IL‐6), and CXCL1 were decreased when treated with 78c compared to controls (Figure 4L), and survival of mice was significantly improved with 78c intervention at 3 and 6 h after modeling (Figure 4M). Besides, histopathology of lung tissues of mice with sepsis revealed inhibition of CD38 at 3 h after modeling, which reduced CLP‐induced inflammatory cell infiltration, as well sepsis‐caused kidney injury was mitigated (Figure 4N,O). Using flow cytometry to analyze the composition of immune cells in lung and kidney tissues, we found that the number of monocytes/macrophages, particularly CD38^high^ monocytes/macrophages, was significantly reduced in the lungs and kidneys of CLP mice after CD38 inhibition (CD38i) (Figure 4P,Q, Figure S6A–D, Supporting Information). Immunofluorescence results further confirmed this finding (Figure S6E, Supporting Information). These results revealed that sepsis‐induced inflammatory responses depended on CD38^high^ monocytes and inhibition of CD38 significantly reduced the multiple organ damage caused by CLP‐induced sepsis.

### 2.7 CD38^high^ Monocytes Exhibited Hyperactivated Glycolysis Properties

**Figure 5:**
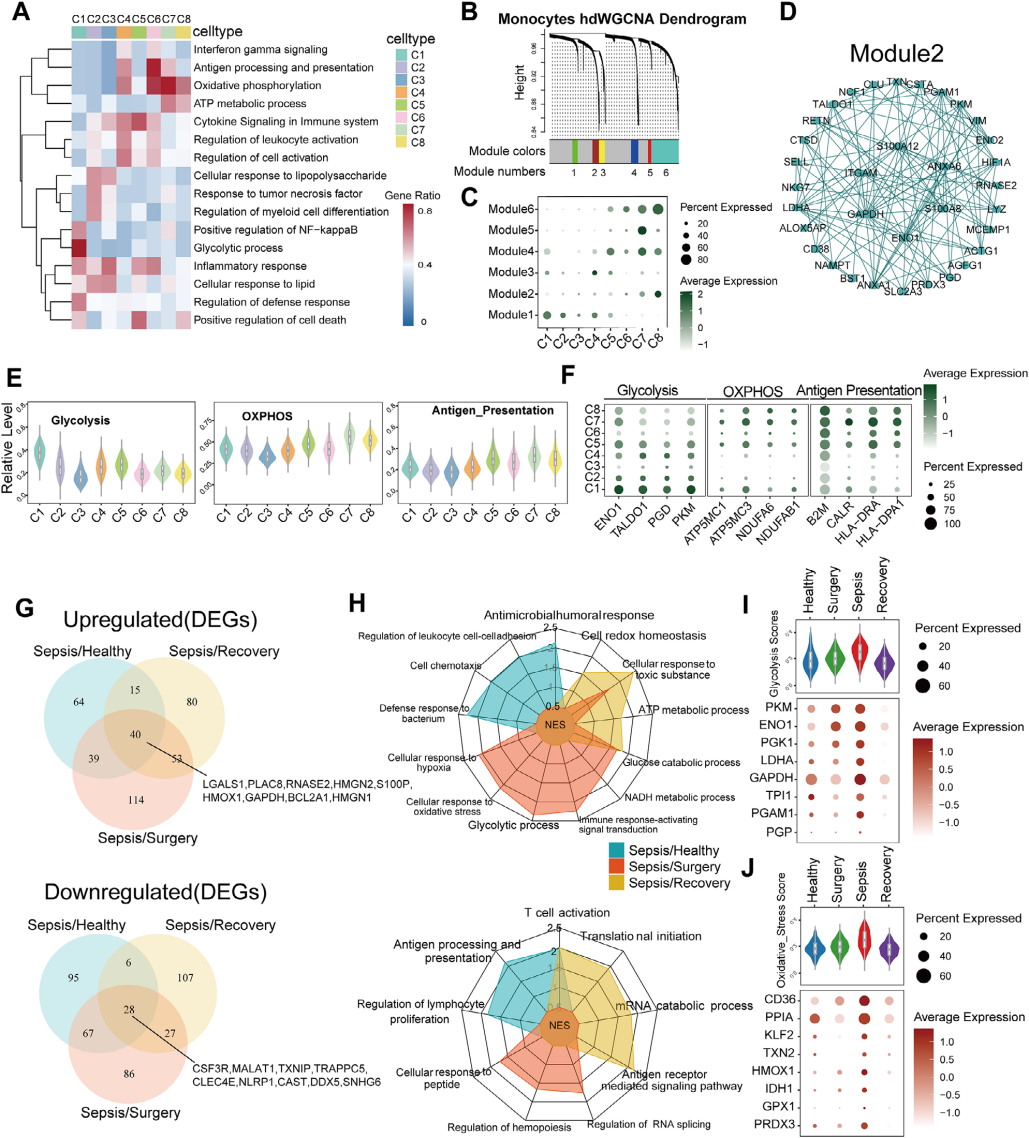
CD38^high^ monocytes exhibited hyperactivated glycolysis properties. A) Gene ontology analysis of cluster‐based DEGs for each subcluster. Selected Gene ontology terms with Benjamini–Hochberg‐corrected *p* < 0.05(one‐sided Fisher's exact test) are shown. B) Dendrogram identifying six hdWGCNA modules in monocytes. C) Dot plot for enrichment of modules in different monocytes subclusters. D) Network plot showing top hub genes for module 2. E) Violin plot showing expression of glycolysis score, oxidative phosphorylation score, and antigen presentation score in each subcluster of monocytes. F) Dot plot of average normalized expression of representative genes in glycolysis signature, oxidative phosphorylation signature, and antigen presentation signature. G) Venn plot showing the number of shared upregulated (upper) and downregulated (lower) DEGs in C1 monocytes between the Sepsis and Healthy groups (Sepsis/Healthy), the Sepsis and Surgery groups (Sepsis/Surgery), the Sepsis and Recovery groups (Sepsis/Recovery). H) Radar plot showing the representative result of GSEA analysis of upregulated (upper) and downregulated (lower) DEGs between different groups in C1 monocytes. The length represents the NES value of the indicated pathway. NES, normalized enrichment score. I) Gene set score analysis of glycolysis pathway in C1 monocytes of different groups (upper), and dot plot of average normalized expression of genes in glycolysis pathway (lower). J) Gene set score analysis of oxidative‐stress pathway in C1 monocytes of different groups (upper), and dot plot of average normalized expression of genes in oxidative‐stress pathway (lower).

We conducted gene ontology (GO) enrichment analysis^[^ ^14^ ^]^ with the signature genes of CD38^high^ monocytes (**Figure** **5**A). Notably, CD38^high^ C1 monocytes‐related genes were significantly enriched in glycolytic process. Moreover, the top hub genes for CD38^high^ C1 monocytes included several glycolysis‐related genes, such as *ENO1*, *LDHA*, *SLC2A3*, *PKM*, and *HIF1A*, were identified by single‐cell weighted gene co‐expression network analysis (hdWGCNA)^[^ ^15^ ^]^ (Figure 5B–D). We investigated the glycolysis signature, oxidative phosphorylation signature, and antigen presentation signature across different subclusters and found that glycolysis activities were significantly higher in CD38^high^ C1 monocytes (Figure 5E,F). Comparing gene expression patterns of CD38^high^ C1 monocytes between the groups in detail, Gene Set Enrichment Analysis (GSEA)^[^ ^16^ ^]^ based on DEGs in different groups revealed that up‐regulated DEGs in the Sepsis/Healthy were mainly associated with defense response to bacterium, whereas up‐regulated DEGs in the Sepsis/Surgery were associated with cellular response to oxidative stress, and glycolytic process (Figure 5G,H). CD38^high^ C1 monocytes of the Sepsis group highly expressed oxidative stress related genes and glycolysis signature related genes, such as *ENO1*, *PKM*, *PGK1*, and *LDHA*, which were key glycolytic enzymes (Figure 5I,J). The above results indicated that CD38^high^ monocytes exhibited hyperactivated glycolysis properties.

### 2.8 CD38‐NAD^+^‐HIF‐1α Axis Induce Metabolic Rewiring of Monocytes in Sepsis

**Figure 6:**
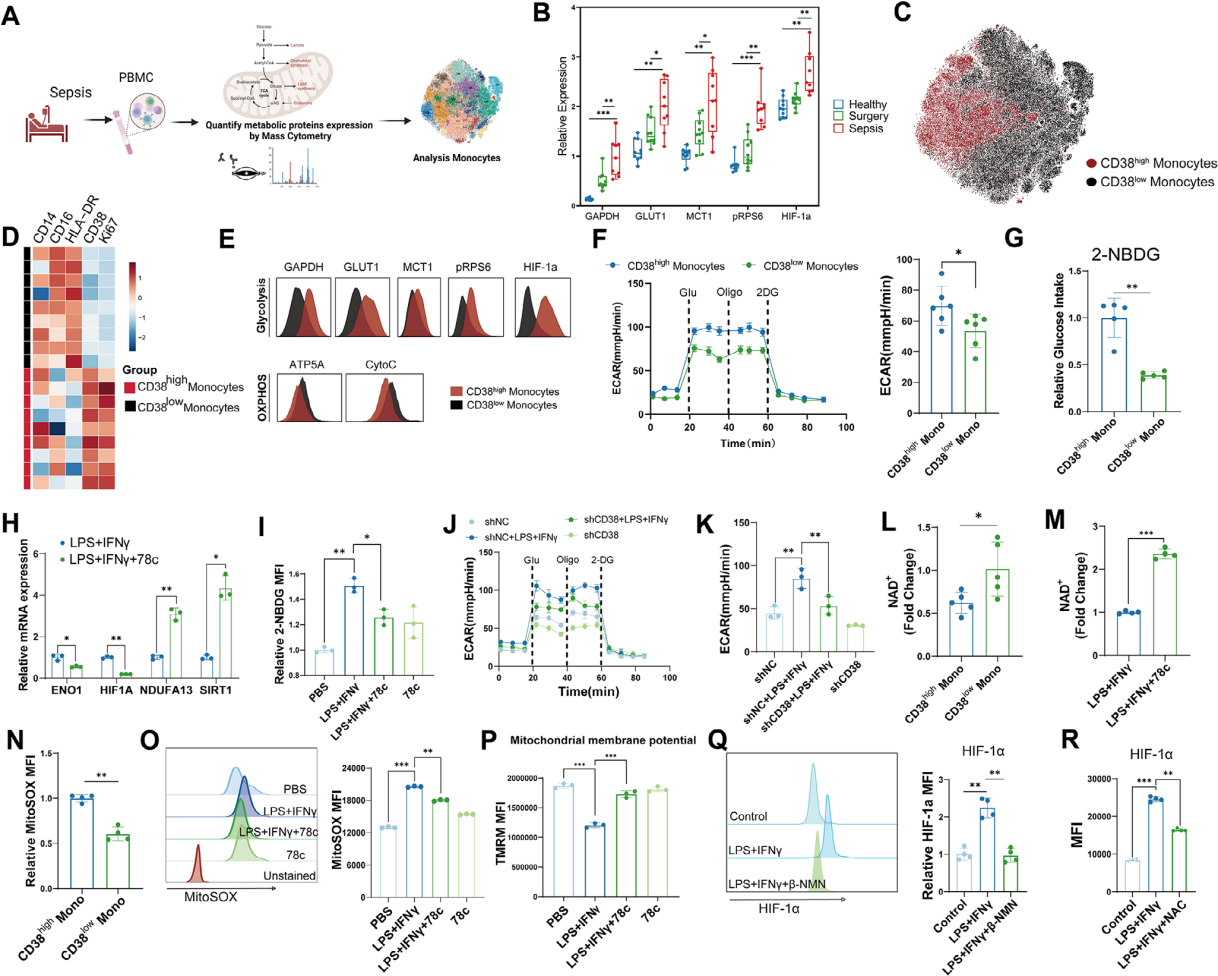
CD38‐NAD^+^‐HIF‐1α axis induce metabolic reprogramming of monocytes in sepsis. A) Schema depicting the workflow for the CD14^+^ monocytes for mass cytometry detecting metabolic protein. B) Boxplot showing expression levels of metabolic proteins in monocytes across different groups (*n* = 9 per group). Statistical significance was determined by a two‐sided, unpaired Wilcoxon rank‐sum test. *p < 0.05, **p < 0.01, ***p < 0.001, ****p < 0.0001. All boxplots show median, first and third quartiles; whiskers 1.5× interquartile range. C) t‐SNE projection for CD38^high^ and CD38^low^ monocytes. D) Functional and phenotypic median expression profiles for CD38^high^ and CD38^low^ monocytes from sepsis (*n* = 9 per group). E) Expression profiles of metabolic proteins in CD38^high^ and CD38^low^ monocytes from sepsis. F) Extracellular acidification rate (ECAR) of CD38^high^ and CD38^low^ monocytes from sepsis analyzed by Seahorse (*n* = 6 per group, independent experiments). G) Glucose uptake assays of CD38^high^ and CD38^low^ monocytes from sepsis (*n* = 5 per group, independent experiments). H) qPCR validation analysis of the representative genes of primary monocytes normalized to *ACTB* (*n* = 3 per group, independent experiments). I) Glucose uptake assays of CD14^+^ monocytes with different treatments (*n* = 3 per group, independent experiments). J) ECAR of CD14^+^ monocytes analyzed by Seahorse. K) Adjacent bar showing ECAR levels after glucose addition (*n* = 3 per group, independent experiments). L) NAD^+^ levels of CD38^high^ and CD38^low^ monocytes from sepsis were measured to quantify CD38 activity (*n* = 5 per group, independent experiments). M) NAD^+^ levels of CD14^+^ monocytes were measured to quantify CD38 activity (*n* = 4 per group, independent experiments). N) Mitochondrial superoxide (MitoSOX) production was assessed by flow cytometry on CD38^high^ and CD38^low^ monocytes from sepsis (*n* = 4 per group, independent experiments). O) MitoSOX production was assessed by flow cytometry on primary CD14^+^ monocytes (*n* = 3 per group, independent experiments). P) Measurement of mitochondrial membrane potential of monocytes with Tetramethylrhodamine, methyl ester (TMRM) (*n* = 3 per group, independent experiments). Q) Expression levels of HIF‐1α were assessed by flow cytometry on primary CD14^+^ monocytes with supplement of β‐NMN (*n* = 4 per group, independent experiments). R) Expression levels of HIF‐1α were assessed by flow cytometry on primary CD14^+^ monocytes with supplement of NAC (*n* = 4 per group, independent experiments). All data are representative of at least two independent experiments performed in triplicate. Error bars represent mean ± SD.*p < 0.05; **p < 0.01; ***p < 0.001; ****p < 0.0001; ns, no significant difference (*p* > 0.05). Two‐tailed unpaired Student's *t*‐test was used in F, G, H, L, M, N; One‐way ANOVA and Tukey post hoc tests for B, I, K, O, P, Q, R.

We next characterized the hyperglycolysis programs of CD38^high^ monocytes in clinical samples at the single‐cell protein level by mass cytometry (**Figure** **6**A), and found that glycolysis‐related proteins were highly expressed in the Sepsis group (Figure 6B,C). Especially, CD38^high^ monocytes expressed elevated levels of GLUT1 and GAPDH, while the expression of key components of oxidative phosphorylation (OXPHOS) including cytochrome c (CytoC) and ATP synthase (ATP5a), were significantly decreased in CD38^high^ monocytes (Figure 6D,E). Moreover, we next assessed extracellular acidification rate (ECAR) of CD38^high^ and CD38^low^ monocytes of sepsis patients isolated by flow cytometry (FACS), and found CD38^high^ monocytes had a higher level of basal glycolysis (Figure 6F), which was also proved by the assessment of glucose uptake capacity (Figure 6G). The alterations of representative genes were further verified by qPCR (Figure 6H).

CD38, a nicotinamide adenine dinucleotide (NAD^+^) hydrolase that inversely correlates to NAD^+^ levels.^[^ ^17^ ^]^ The role of CD38 in the hyperactivated glycolysis properties of CD38^high^ monocytes remains unclear. Inhibition of CD38 diminished LPS + IFN‐γ induced glucose uptake in monocytes (Figure 6I), which was further validated by the decreased ECAR in *CD38* stable knockdown cultured monocytes THP‐1 cells (Figure 6J,K). Additionally, we observed a significant elevation in NAD^+^ levels in CD38^low^ monocytes and CD38‐inhibited primary monocytes (Figure 6L,M). During metabolic reprogramming, NAD^+^ consumption results in mitochondrial dysfunction.^[^ ^18^ ^]^ We observed that mitochondrial ROS produced by CD38^high^ monocytes significantly elevated as measured by MitoSOX (Figure 6N). Blockade of CD38 reduced LPS+IFN‐γ induced mitochondrial ROS production (Figure 6O). Upon inhibition of CD38, the depolarization of mitochondrial membrane in monocytes was re‐established (Figure 6P). These results suggested that the mitochondrial dysfunction induced by NAD^+^ consumption depends on CD38.

It is evident that mitochondrial dysfunction induced hypoxia‐inducible factor‐1α (HIF‐1α) stability, which is a strong inducer between NAD^+^ consumption and glycolysis.^[^ ^19^ ^]^ Treatment of LPS and IFN‐γ induced monocytes with the supplement of β‐nicotinamide mononucleotide (β‐NMN), an NAD^+^ precursor, and antioxidants thiol reductant *N*‐acetyl cysteine (NAC), was sufficient to inhibit HIF‐1α expression and stabilization (Figure 6Q,R).

To verify whether this conclusion is conservative in septic mice, we re‐analyzed a published single‐cell transcriptome data of isolated mouse monocytes.^[^ ^20^ ^]^ Comparing the expression distribution, we found that *Cd38* and a series of glycolysis‐related genes were significantly increased in the LPS‐induced Mono1 subpopulation (Figure S7A–C, Supporting Information). We next isolated mouse peripheral monocytes and found that sepsis‐induced CD38 activation was significantly reduced with 78c intervention at 3 h after modeling (Figure S7D, Supporting Information), and observed the NAD^+^ levels were rescued, whereas the expression levels of HIF‐1a were significantly reduced with 78c intervention at 3 and 6 h after modeling (Figure S7E,F, Supporting Information), which demonstrated the role of CD38‐NAD^+^ ‐HIF‐1α‐glycolysis axis in monocytes of septic mice.

### 2.9 Positive Feedback Regulation of CD38^high^ Monocytes in Sepsis through Glycolytic Metabolite Methylglyoxal

**Figure 7:**
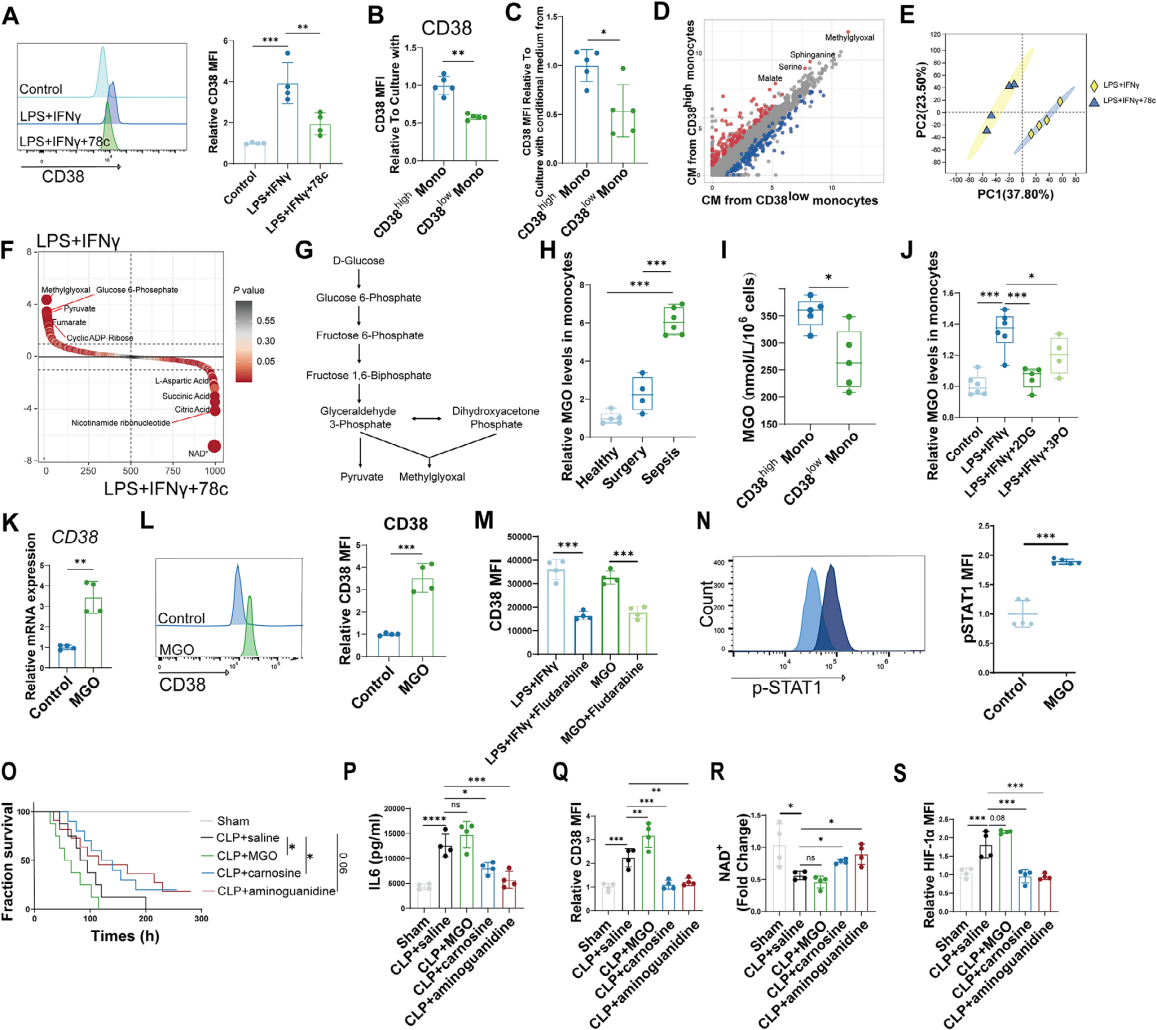
Positive feedback regulation of CD38^high^ Monocytes in sepsis through glycolytic metabolite methylglyoxal. A) Expression levels of CD38 were assessed by flow cytometry on primary CD14^+^ monocytes with inhibition of CD38 (*n* = 4 per group, independent experiments). B,C) Mean fluorescence intensity of CD38 in primary CD14^+^ monocytes in co‐cultures of CD38^high^ or CD38^low^ monocytes from whole blood of patients with sepsis (B) (*n* = 5 per group, independent experiments) and supplemented with the conditional mediums from CD38^high^ or CD38^low^ monocytes from sepsis patients (C) (*n* = 5 per group, independent experiments). D) Volcano plot showing the differential metabolites in the conditioned medium of CD38^high^ monocytes and CD38^low^ monocytes (*n* = 4 per group, independent experiments). E) The principal component analysis (PCA) plot showing the results of metabolites identified by unbiased metabolomics in human primary CD14^+^ monocytes (*n* = 4 per group). F) Dot plot showing the relative levels of metabolites in monocytes with different treatments. G) Schematic representation of the steps of glycolysis and MGO production. H) MGO quantification in monocytes from different groups (Healthy, *n* = 5; Surgery, *n* = 4; Sepsis, *n* = 6). I) MGO quantification in CD38^high^ and CD38^low^ monocytes from sepsis patients (*n* = 5 per group, independent experiments). J) MGO quantification in monocytes with the inhibition of glycolysis using 2DG and 3PO (Control, *n* = 6; LPS + IFNγ, *n* = 6; LPS + IFNγ + 2DG, *n* = 5; LPS + IFNγ + 3PO, *n* = 4). K) mRNA expression levels of CD38 were assessed on primary CD14^+^ monocytes with the stimulation of MGO (*n* = 4 per group). L) Expression levels of CD38 were assessed by flow cytometry on primary CD14^+^ monocytes with the stimulation of MGO (*n* = 4 per group). M) Expression levels of CD38 were assessed by flow cytometry on primary CD14^+^ monocytes with the inhibition of pSTAT1 (*n* = 4 per group). N) Expression levels of pSTAT1 were assessed by flow cytometry on primary CD14^+^ monocytes with the stimulation of MGO (*n* = 5 per group). O) Survival rates of sepsis model mice (*n* = 9 per group; log‐rank test [Mantel–Cox]). P) IL6 levels in serum from septic mice measured by ELISA (*n* = 4 per group). Q) Expression levels of CD38 were assessed by flow cytometry on mouse CD14^+^ monocytes with different treatments (*n* = 4 per group). R) Measurement of NAD^+^ levels of mouse monocytes from sepsis model (*n* = 4 per group). S) Expression levels of HIF‐1a were assessed by flow cytometry on mouse CD14^+^ monocytes with different treatments (*n* = 4 per group). All data are representative of at least two independent experiments performed in triplicate. Error bars represent mean ± SD.*p < 0.05; **p < 0.01; ***p < 0.001; ****p < 0.0001; ns, no significant difference (*p* > 0.05). Two‐tailed unpaired Student's *t*‐test was used in B, C, I, L, N; One‐way ANOVA and Tukey post hoc tests for A, H, J, M, P, Q, R, S.

To explore the underlying reasons for enrichment of CD38^high^ monocytes in sepsis, we re‐analyzed our RNA‐seq data and found a significant reduction in *CD38* expression in monocytes upon CD38 inhibition (Figure 4D). This finding suggests that the role of CD38 in monocytes may involve a positive feedback regulation of CD38 expression. It was evidenced that inhibiting CD38 diminished LPS + IFN‐γ induced CD38 elevation in monocytes (**Figure** **7**A). Given the hyperactivated glycolysis property of CD38^high^ monocytes, we hypothesized that metabolites produced by CD38^high^ monocyte further promote the expression of CD38. Thus, we collected conditioned medium of CD38^high^ and CD38^low^ monocytes of sepsis, which were co‐cultured with CD14^+^ monocytes respectively. CD38^high^ monocytes‐derived conditioned medium significantly increased the expression of CD38 in CD14^+^ monocytes (Figure 7B), as further confirmed by co‐culturing with CD38^high^ and CD38^low^ monocytes directly (Figure 7C).

Using an unbiased metabolomics analysis of conditioned medium derived from CD38^high^ and CD38^low^ monocytes, we identified several metabolites that were significantly upregulated in the CD38^high^ monocyte group, with the increase in methylglyoxal (MGO) being particularly notable (Figure 7D). Furthermore, post‐treatment with 78c, metabolomic analysis showed a significant reduction in methylglyoxal (MGO) levels within monocytes (Figure 7E,F). MGO is a highly reactive carbonyl species, produced endogenously from the spontaneous degradation of glyceraldehyde‐3‐phosphate and dihydroxyacetone phosphate during glycolysis (Figure 7G). Consistently, concentrations of MGO in monocytes in patients with sepsis were significantly increased (Figure 7H). Moreover, we observed an increase in the concentrations of MGO in CD38^high^ monocytes (Figure 7I). Given the non‐glycolytic sources of methylglyoxal, we used 2‐Deoxy‐D‐glucose (2DG) and 3PO to block the glycolysis pathway in monocytes stimulated with LPS and IFN‐γ and found that the concentrations of MGO were significantly reduced (Figure 7J), which proved that the elevated concentrations of MGO in CD38^high^ monocytes were mainly derived from glycolysis pathway.

We next directly treated monocytes with MGO and found the expression of CD38 in monocytes significantly elevated (Figure 7K,L). It was reported that STAT1 pathway mediated CD38 upregulation on myeloma cells and endothelial cells.^[^ ^21^ ^]^ We found that the expression of CD38 in monocytes stimulated with LPS and IFN‐γ or MGO was significantly reduced by fludarabine as a STAT1 specific inhibitor (Figure 7M), and MGO treatment also directly activated STAT1 phosphorylation (Figure 7N), which suggesting MGO upregulates STAT1 phosphorylation and facilitates CD38 expression. In addition to MGO, we also investigated the effect of several metabolites and cytokines in expression of CD38. We found that treating monocytes with various concentrations of NAD^+^ metabolites (ADPR and cADPR), glycolysis metabolite lactate, and cytokines (IL6+TNFα+IL1β) did not lead to an upregulation of CD38 expression (Figure S8A–C, Supporting Information). Furthermore, upon re‐examining the differential metabolites in the media, we observed no significant change in CD38 expression following treatment with malate, serine, and sphinganine (Figure S8D,E, Supporting Information).

**Figure 8:**
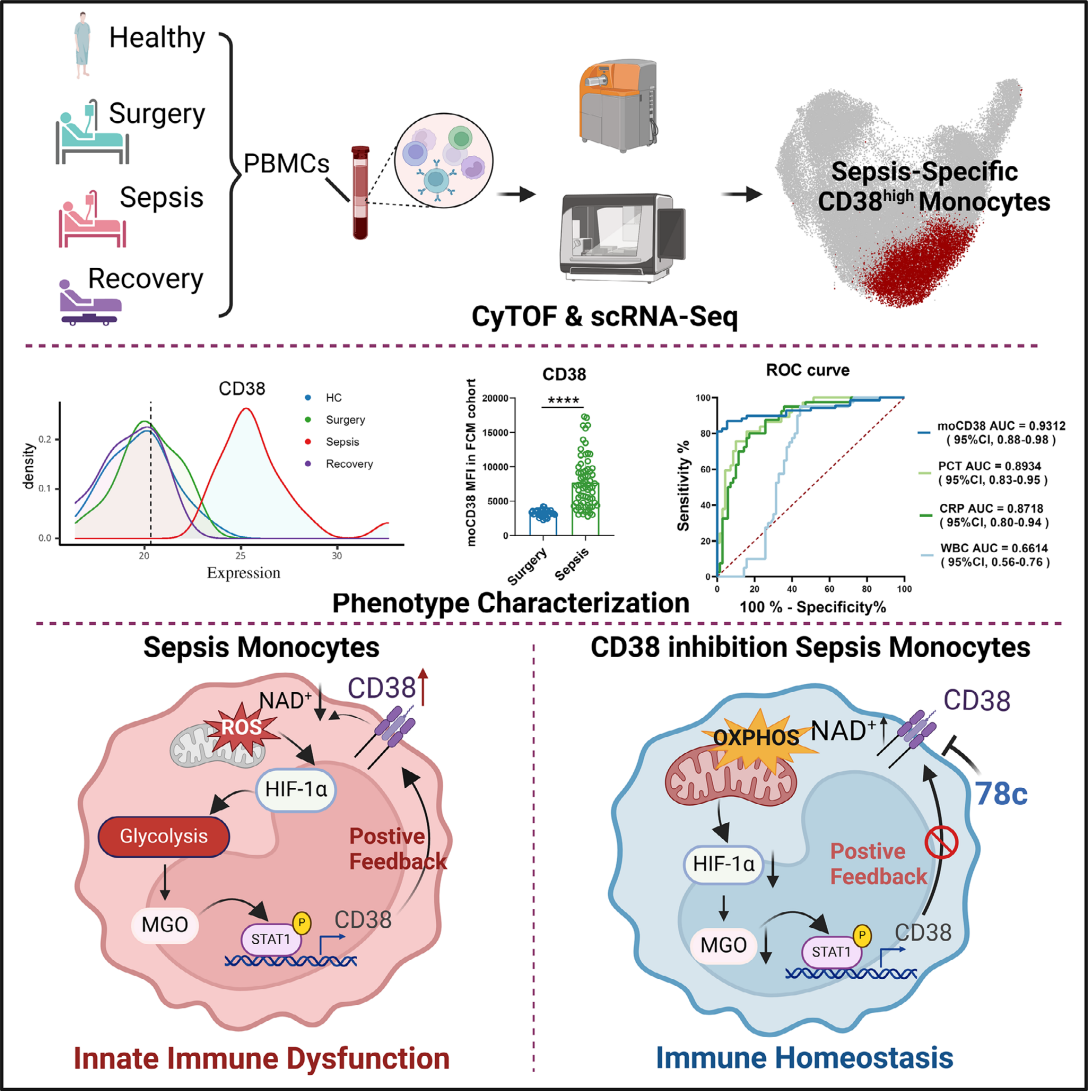
Schematic illustration. A schematic illustration showing that novel biomarker CD38^high^ monocytes mediated the CD38‐NAD^+^‐HIF1‐α/glycolysis/MGO loop to promote the activation of monocytes and exacerbated sepsis‐induced immune dysfunction.

Similarly, the survival of sepsis mice was significantly reduced with MGO administration and improved with two potent MGO scavengers (carnosine and aminoguanidine) intervention (Figure 7O). The levels of IL6 in plasma were also significantly reduced after MGO clearance (Figure 7P). Moreover, both carnosine and aminoguanidine significantly decreased the expression of CD38 and levels of NAD^+^ in mouse monocytes, whereas additional MGO supplementation increased the expression of CD38 (Figure 7Q,R), and the expression levels of HIF‐1a in monocytes were significantly reduced by removing MGO in vivo (Figure 7S, Supporting Information). Collectively, these results provided evidence of our hypothesis that the CD38‐NAD^+^‐HIF1‐α/glycolysis/MGO loop promoted the activation of monocytes and therefore exacerbated sepsis‐induced immune dysfunction (**Figure** **8**).

## 3 Discussion

Excessive activation of monocytes may be the cornerstone of systemic immune dysfunction in sepsis.^[^ ^22^ ^]^ Detailed knowledge of the cellular processes of sepsis monocytes is essential to identify early diagnostic signatures and personalized immune‐based therapeutic targets.^[^ ^1^, ^23^ ^]^ Recent studies utilizing Single‐cell RNA‐seq of PBMCs from septic patients have disclosed the expansion of an immature bone‐marrow‐derived monocyte state (MS1).^[^ ^11c^ ^]^ HLA‐DR^low^S100A^high^ monocytes were also prominently enriched in septic patients and exerted immunosuppressive function.^[^ ^24^ ^]^ And fresh whole‐blood single‐cell multiomic atlas exhibited a distinct immature IL1R2 Neu population. Consistent with these findings, our data systematically compared the composition of peripheral immune cells among sepsis patients, sterile inflammation patients, mild infectious patients, and sepsis recovery patients using scRNA‐seq and single‐cell mass cytometry to identify that novel CD38^high^ monocytes specifically accumulated within the first 24 h of sepsis. Most importantly, our findings demonstrated that CD38^high^ monocytes not only could serve as a diagnostic biomarker for sepsis, but also represented a potential therapeutic target to reduce inflammatory response in combating sepsis.

An ideal biomarker had features of high sensitivity and specificity, and can be identified by fully automated technology with low production costs.^[^ ^25^ ^]^In our study, though novel CD38^high^ monocytes were identified by high cost scRNA‐seq and CyTOF, it was excited to observe that CD38^high^ monocytes were detectable by lower cost flow cytometry, and with an AUC surpassing that of CRP and PCT to discriminate Sepsis group and Surgery group, which suggested its diagnostic values to distinguish sepsis and non‐infectious systemic inflammation. Bacterial microorganisms were identified in 79.9% of infection among patients in intensive care units.^[^ ^25^ ^]^ Our results demonstrated the CD38^high^ monocytes may be beneficial for clinician discriminating sepsis from noninfectious critical illness and predicting outcomes of bacterial sepsis using flow cytometry techniques available in routine laboratories. In our subgroup analysis, the abundance of CD38^high^ monocytes showed a positive correlation with the severity of BS subgroup bacterial sepsis (e.g., CRP, APACHE II scores) and 28‐day mortality. This finding holds significance, as existing biomarkers of host immune response typically only indicate sepsis risk stratification.^[^ ^26^ ^]^ Meanwhile, we observed differences in the predictive value of moCD38 between the sepsis group and the BS subgroup. We speculate that etiological heterogeneity may be an important reason. The sepsis group includes various pathogens (bacteria, fungi, and other infections), and the differences in their pathophysiological mechanisms may be the cause of the inconsistent results between the two groups. Moreover, differences in treatment strategies (such as antibiotic selection and supportive therapy) may affect the clinical indicator responses and prognoses of patients with different sepsis subtypes.

Early antibiotics treatment and effective anti‐inflammatory control is a critical element of treatment for sepsis.^[^ ^26^ ^]^ CD38, as a type II transmembrane glycoprotein with metabolic enzymatic function, can catalyze NAD^+^ to nicotinamide and other metabolites.^[^ ^27^ ^]^ While CD38 has been extensively studied as a targeted therapy for multiple myeloma,^[^ ^28^ ^]^ recent research demonstrated that CD38 inhibition enhanced the effector function of CD8^+^ T cells, reversed mitochondrial defects, and improved viral clearance.^[^ ^29^ ^]^The roles of CD38 in primary monocytes of sepsis were not fully illustrated till now. The abundances of CD38^high^ monocytes were significantly higher in the first 24 h of sepsis than that of 7 days after (Recovery group), indicating early CD38^high^ monocytes targeted intervention may be beneficial. Subsequently, our findings revealed that CD38 inhibition and deletion in monocytes significantly dampened the inflammatory response and oxidative stress. In a CLP sepsis mouse model, *Cd38*‐kncokout and pharmaceutical early intervention of CD38 reduced the disease phenotype of sepsis mouse model, making it a promising therapeutic target to treat patients with sepsis.

Moreover, alterations in metabolism of monocytes during sepsis are implicated in the diverse immune responses observed in sepsis.^[^ ^30^ ^]^ Identifying the interplay between metabolism and disease is essential for sepsis treatment. The metabolic reprogramming of monocytes to glycolysis during sepsis is an indispensable component of initiation of the host defense response, with intermediate metabolites of glycolysis also playing a role in innate immune dysregulation.^[^ ^31^ ^]^ We observed that CD38^high^ monocytes exhibited higher glycolytic activity, which depended on HIF‐1α activation with the key mediators of the cellular metabolism.^[^ ^32^ ^]^ Moreover, the reduction in NAD+ directly leads to insufficient production of NADH, a critical electron donor for the electron transport chain, thereby hindering its normal function and inducing overall mitochondrial dysfunction to potentially activate apoptotic signaling pathways. These chain reactions weaken crucial components of oxidative phosphorylation at the molecular level. Similarly, in our study, NAD^+^ consumption induced mitochondrial insufficiency elicited the stabilization of HIF‐1α, which in turn triggered the metabolic shift to aerobic glycolysis, thus CD38‐NAD+‐HIF‐1α axis was found to induce metabolic rewiring of monocytes in sepsis. Maintaining the stability of energy metabolism in monocytes plays an important role in sepsis.^[^ ^33^ ^]^

Surprisingly, we also observed a significant reduction in CD38 expression upon its inhibition, and MGO was identified as a key mediator in this positive feedback loop. MGO is endogenously produced as a spontaneous degradation product of metabolites within the glycolytic pathway.^[^ ^34^ ^]^ Importantly, hyperglycemia is a clinical characteristic of sepsis patients.^[^ ^35^ ^]^ In our clinical statistics, we also found that the blood glucose levels of patients with bacterial sepsis were significantly higher than those of surgical patients (Table S1, Supporting Information). However, the consequences of elevated blood glucose, particularly regarding MGO production and its potential role in the development and progression of sepsis, remain largely unexplored. We found that the massive production of MGO promotes the elevation of CD38 through pSTAT1 activation via a positive feedback loop, which supported the conceptualization of the progression of sepsis as a process of amplification originating from an initial perturbation, instead of following a linear cascade. The similar results were further observed in mortality among CLP mice, indicating that MGO synthesis is a crucial biochemical pathway induced in sepsis, contributing to cellular dysfunction and inflammation.

Our study provided a comprehensive cell atlas of sepsis, as well as specific insight into a novel biomarker of sepsis with a unique disease metabolic phenotype, which provide a robust and effective tool to better illustrate the heterogeneous immune processes of sepsis. In addition, our study has revealed a CD38‐NAD^+^‐HIF‐1α/glycolysis/MGO positive feedback loop that exacerbates monocytes activation and dysfunction in sepsis. However, there are some limitations of this study. Firstly, given the heterogeneity of sepsis, a unified pathogenesis among patients of different ages and disease subtypes remains unclear. The limited number of patients included in the discovery and validation cohorts restricts the generalizability of the results to a broader population. Secondly, the absence of sampling at multiple time points in sepsis patients hampers the temporal resolution of the analysis. The immune response in sepsis patients is highly dynamic, and additional time points might reveal further pathophysiological mechanisms that distinguish sepsis from aseptic inflammation. Moreover, CD38^high^ monocytes show significant potential as a sole indicator for distinguishing sepsis from other patients. However, a single indicator might not fully encapsulate this cell population, leaving room for technical optimization in the future, which could involve integration with existing clinical scoring systems and exploration of other subpopulation‐related proteins. It is still unknown whether CD38^high^ monocyte can distinguish between bacterial, viral, fungal, etc. infections, and its predictive value for viral and fungal sepsis is not yet clear. Regarding in vivo function, we have determined that CD38^high^ monocytes induce metabolic disorder and inflammation by modulating glycolysis, although their specific cellular functions remain partially unclear, especially the role of migrating CD38^high^ monocytes in sepsis‐related tissue damage.

## 4 Experimental Section

### Study Design

The study was designed to determine the diagnostic and functional role of CD38^high^ monocytes in sepsis. Cohorts study design included scRNA‐seq discovery cohort, CyTOF validation cohort 1 and FC validation cohort 2 (Figure 1A). ScRNA‐seq discovery cohort comprised 3 patients in each group, those were patients with sepsis (Sepsis), patients undergoing cardiac surgery considered as sterile inflammation control (Surgery), patients recovered from sepsis on the seven days (Recovery), and age‐ and sex‐matched healthy donors (HC), combined with 14 sepsis patients and 10 mild infectious patients (urinary‐tract infectious patients without organ dysfunction) sourced from public databases. CyTOF validation cohort1 included 35 Sepsis patients, 20 Surgery patients, 20 HC volunteers, 12 Mild patients (patients in the Mild group should be diagnosed as infected cases, but the SOFA score increases by less than 2), and 11 Recovery patients. 102 Sepsis patients, 53 Mild patients, and 98 Surgery patients were included in FC validation cohort2. Sepsis patients were diagnosed with the clinical criteria of Sepsis‐3. The inclusion criteria for Sepsis group included: 1) Participants ≥18 years old; 2) Participants had to be diagnosed with infections and SIRS score ≥2 within 24 h; 3) △SOFA score ≥2. Patients who were younger than 18 years of age, who were pregnant or breast‐feeding; who were diagnosed with malignancy, organ transplantation, HIV infection, or autoimmune disease; or who were using immunosuppressive medication were excluded from the cohort study. The sepsis patients were further classified into bacterial sepsis (BS) and other pathogen sepsis (OS) subgroups based on the types of pathogenic microorganisms. The BS subgroup indicated that bacterial pathogens, such as *Klebsiella pneumoniae* and *Pseudomonas aeruginosa*, were responsible for causing sepsis at the infection site. In contrast, pathogens identified at the source sites of sepsis in the OS subgroup included fungi, viruses, or mixed infections involving bacteria.

Blood samples were obtained within 24 h of the diagnosis of sepsis and within 24 h after surgery. And blood samples were collected again on the seventh day after the patient recovered from sepsis. Whole peripheral blood samples were drawn in EDTA‐coated anticoagulant tubes. This observational study was approved by the human research ethics committees at the First Affiliated Hospital, Zhejiang University School of Medicine, Hangzhou, China (ID: IIT20220071B and ID: IIT20230282B). The donors (or their relatives) of the blood samples used in this study provided written informed consent.

Functional studies were performed in vivo in sepsis mice models and in vitro in human and mouse derived monocytes to determine whether CD38 is a candidate therapeutic target for treating sepsis and to understand the underlying regulatory mechanisms by which CD38^high^ monocytes modulates the metabolic phenotype and mechanism. For all in vivo experiments, mice were randomly assigned to experimental groups. Animals were housed in a specific pathogen‐free facility maintained below 22 °C and 55% humidity under a 12‐h light‐dark cycle and free access to food and water in the Laboratory Animal Center. The animal experimental protocols were approved by the Review Committee of Zhejiang University School of Medicine (ID: ZJU20230076) and were in compliance with institutional guidelines.

### Mass Cytometry Profiling

The single‐cell suspensions were barcoded using ^194^Pt for distinguishing live/dead cells, then treated with block mix for 20 min and stained with surface marker staining mix for 30 min on ice. After washing, resuspension, and centrifugation, these samples were incubated with DNA Intercalator ^191^Ir and ^193^Ir overnight at 4 °C. Before running on mass cytometry, unique sample barcoding was performed on individual samples for 30 min. Finally, single cells were resuspended into deionized (DI) water, after the tuning and quality control (QC) procedures, the calibration beads were mixed with barcoded samples and collected with CyTOF with 500 events per second. In total, nearly 20 million immune cells were collected after preprocessing for downstream analyses. Differential abundance analysis between different groups was performed using code from the diffcyt package (version 1.8.8) with the option testDA_edgeR.

### scRNA‐seq Library Preparation and Sequencing

Single‐cell suspensions from PBMC samples were obtained and prepared to a concentration of 700–1200 viable cells µL^−1^. Single‐cell transcriptome libraries were generated using the 10X Genomics Chromium. Single‐cell transcriptome libraries were also generated using the MobiNova single‐cell library construction system (MobiDrop) for validation. The libraries were sequenced with illumine sequencing platform.

### Measurement of Extracellular Acidification Rate (ECAR)

ECAR was measured by Seahorse XF96 analyzer (Agilent Technologies). THP‐1 monocytes with or without CD38 knockdown were seeded on XF96 plates at 1 × 10^5^ cells per well one day prior to the XF assay. On the day of assay, the medium was replaced with assay medium composed of XF Base Medium without phenol red, adjusted to pH 7.4 and incubated at 37 °C without CO_2_ 45 min prior to XF assay.

### scRNA‐seq Analysis

Raw scRNA‐seq reads were barcode‐deduplicated and aligned to the hg38 reference genome using Cell Ranger v.3.1.0, yielding sparse digital count matrices, which were analyzed to identify cell types and cellular states using Seurat v.4.0.0. Outlier cells were identified and removed based on the following criteria: 1) >20% mitochondrial content or 2) cells with less than 100 or greater than 3000 expressed genes, depending on sample‐level distributions. The top 2000 highly variable genes (HVGs) from the normalized expression matrix were identified, centered, and scaled before performing the principal component analysis (PCA) based on these HVGs. The potential doublets were removed using the DoubletFinderpackage (version 2.0.3) of the R. To remove batch effects among experiments and samples, canonical correlation analysis (CCA) was used to process the data and published datasets. Importantly, the batch‐corrected data were only used for PCA and all other steps relying on PCA. All other analyses (e.g., differential expression analysis) were based on the normalized data without batch correction.

### Dimensionality Reduction and Cell‐Type Annotation

The entire data was projected onto two‐dimensional space using UMAP on the top 20 principal components. The FindClusters function (resolution: 0.5) was performed to cluster cells according to co‐expressed features. The FindAllMarkers function with parameters log_2_FC.threshold = 0.5 and test.use =  “wilcox” in Seurat was used to find markers for each of the identified clusters. Clusters were then classified and annotated based on expressions of canonical markers for specific cell types. To identify differentially expressed genes between two clusters, the find.markers function of the Seurat was used with log_2_FC.threshold = 0.5 and test.use =  “Wilcox.”

### Defining Cell State Scores

The AddModuleScore function of the Seurat R package was used to evaluate the module scores for the degree of gene expression programs in single cells. The cell scores were based on the average expression of the genes from the predefined gene set in the respective cells. Gene sets were obtained from the MSigDB database (https://www.gsea‐msigdb.org/gsea/msigdb/). The main signatures defined in this work were Glycolysis(Reactome Pathway Database#R‐MMU‐70171), Oxidative Phosphorylation(GO:000619), Antigen Presentation(GO:0019882), Inflammatory Response(HALLMARK_INFLAMMATORY_RESPONSE), Response To Oxidative Stress(GO:0006979), Cell Chemotaxis(GO:0030593), Phagocytosis(GO:0006911), Tricarboxylic Acid Cycle(GO:0006099), Pentose Phosphate Shunt(GO:0009052), Electron Transport Chain(GO:0022900), and Mitochondria‐mediated ROS Production(GO:1903409).

### GO Enrichment Analysis and Gene Sets Enrichment Analysis

Gene Set Enrichment Analysis (GSEA) and Gene Ontology (GO) functional enrichment (over‐representation) of DEGs at *p* < 0.05 was analyzed using an R package clusterProfiler (version 3.18.1). A cutoff adjusted *p*‐value of 0.05 was used to filter the significant enrichment results.

### Isolation and Culture of Blood Monocytes

Peripheral blood mononuclear cells were isolated from blood samples by centrifugation on Ficoll‐paque Plus. CD14^+^ monocyte isolation kit (Miltenyi Biotec) was used to obtain purified CD14^+^ monocytes. Purified monocytes were cultured in IMDM medium containing 10% (v/v) human serum.

### RNA‐Sequencing and Differentially Expressed Genes Analysis

A total of 3 µg RNA per sample was used as input material for the RNA sample preparation. Sequencing libraries were generated using the NEBNext UltraTM RNA Library Prep Kit for Illumina (NEB, USA) following the manufacturer's recommendations, and index codes were added to attribute sequences to each sample. Libraries were normalized, pooled, and sequenced on a single lane of an Illumina NovaSeq. Poly‐A/T stretches and Illumina adapters were trimmed from the reads using cutadapt, and resulting reads shorter than 30 bp were discarded. The remaining reads were mapped onto 3′ UTR regions (1000 bases) of the Mus musculus, mm10 genome according to Refseq annotations, using STAR with the EndToEnd option and with outFilterMismatchNoverLmax set to 0.05. Deduplication was accomplished by flagging all reads mapped to the same gene and had the same Unique Molecular Identifier (UMI).

Differential expression analysis of two conditions/groups (two biological replicates per condition) was performed using the DESeq2 R package (1.16.1). DESeq2 provides statistical routines for determining the differential expression in digital gene expression data using a model based on the negative binomial distribution. The resulting *p* values were adjusted using the Benjamin and Hochberg approach for controlling the false discovery rate. Genes with an adjusted *p* value <0.05 found by DESeq2 were defined as differentially expressed.

### CD38 Enzymatic Activity Assay (ε‐NAD)

CD38 activity was measured using the nicotinamide 1,N6‐ethenoadenine dinucleotide (ε‐NAD) fluorescence‐based enzymatic assay, as described previously. Primary astrocyte cells were incubated with 30 µm ε‐NAD in an extracellular solution of Hanks′ Balanced Salt solution (HBSS) with Ca^2+^/Mg^2+^. Following a 15‐min incubation, fluorescence was read at *λ*Ex = 300 nm and *λ*Em = 410 nm using a fluorescence microplate reader. Normalized values are presented as CD38 activity.

### Flow Cytometry

PBMCs or monocytes were stained with fluorochrome‐labeled antibodies for the surface CD14 and CD38 markers. The stained cells were immediately analyzed with FlowJo Software. Because the MFls varied among different batches, the MFl was normalized to an internal control each time.

### Methylglyoxal Quantification

The Methylglyoxal assay kit (ab241006) was used to enable the detection of MGO using a set of engineered enzymes and a chromophore. By determining the fixed number of each sample and separating the cell fragmentation products, the MGO level in each sample was detected by the enzyme activity detection method in the kit.

### Lentivirus Infection

The shRNA for human *CD38* was packaged into lentivirus by GenePharma Co., Ltd. (Shanghai, China). THP‐1 cells were transfected with lentivirus carrying shRNA‐CD38(shCD38) or negative control sequences(shNC) for 24 h and then cultured in a new RPMI 1640 medium for 48 h. Stable cells line were screened by 3 µm puromycin for more than 48 h. The knockdown efficiency of CD38 in THP‐1 was detected by qPCR and flow cytometry. The shRNA sequences are listed as follows.

LV3‐CD38:5′‐CCAGAGAAGGTTCAGACACTA‐3′;

LV3‐NC: 5′‐TTCTCCGAACGTGTCACGT‐3′.

### Quantitative Real‐Time PCR

The total RNA of cell was collected by using FastPure Cell/Tissue Total RNA isolation Kit V2 (Vazyme, Nanjing, China) according to the manufacturer's protocol. cDNA was synthesized by using PrimeScript RT reagent Kit with gDNA Eraser according to the manufacturer's protocol from 1000 ng RNA from each sample. Quantitative real‐time PCR was conducted using the ChamQ Universal SYBR qPCR Master Mix on a 7500 fast Real‐Time PCR system. The relative mRNA expression of each gene normalized to reference gene expression was calculated using the ∆∆Ct method.

### Metabolomics

Liquid Chromatograph Mass Spectrometer (LC‐MS/MS) analysis were performed using a UHPLC (1290 Infinity LC, Agilent Technologies) coupled to a quadrupole time‐of‐flight (AB Sciex TripleTOF 6600). For hydrophilic interaction liquid chromatography separation, samples were analyzed using a 2.1 mm × 100 mm Acquity UPLC BEH 1.7 µm column (Waters, Ireland). In both ESI positive and negative modes, the mobile phase contained *A* = 25 mm ammonium acetate and 25 mm ammonium hydroxide in water and *B* = acetonitrile. The gradient was 85% *B* for 1 min and was linearly reduced to 65% in 11 min, and then reduced to 40% in 0.1 min and kept for 4 min, then increased to 85% in 0.1 min, with a 5‐min re‐equilibration period. The ESI source conditions were set as follows: Ion Source Gas1 (Gas1) as 60, Ion Source Gas2 (Gas2) as 60, curtain gas (CUR) as 30, source temperature: 600 °C, IonSpray Voltage Floating ± 5500 V. In MS only acquisition, the instrument was set to acquire over the *m*/*z* range 60–1000 Da, and the accumulation time for the TOF MS scan was set at 0.20 s per spectra. In auto MS/MS acquisition, the instrument was set to acquire over the *m*/*z* range 25–1000 Da, and the accumulation time for the product ion scan was set at 0.05 s per spectra. The product ion scan was acquired using information‐dependent acquisition with high‐sensitivity mode selected. The collision energy was fixed at 35 V with ± 15 eV and the de‐clustering potential was set at ± 60 V.

### Glucose Uptake Assay

CD14^+^ Monocytes were seeded at 5 × 10^6^ per well in 12‐well plates and treated as indicated. The cells were starved for 30 min at 37 °C in Krebs–Ringer bicarbonate solution (KRBH, in mm: 135 NaCl, 3.6 KCl, 0.5 NaH_2_PO_4_, 1.5 CaCl_2_, 2 NaHCO_3_, 10 HEPES, and 0.1% BSA). Then the cells were incubated in KRBH supplemented with 2NBDG (60 µm) at 37 °C for 15 min. After collected and centrifuged, cells were resuspended in KRBH for FACS analysis.

### Superoxide Detection Assay

Monocytes from healthy donors were given treatments as indicated in Section 2. Thereafter, these cells were labeled with 5 µm MitoSOX reagent as per manufacturer protocol. Generation of superoxide by monocytes was detected by flow cytometry.

### CLP Model

CLP‐induced sepsis was performed in C57BL/6 (WT, wild‐type) mice. At different time points before and after CLP modeling, a group of mice received an intraperitoneal injection of 78c (5mg kg^−1^) or saline vehicle. Laparotomy was performed to isolate the cecum of mice anesthetized with isoflurane. Approximately two‐thirds of the cecum was ligated with a 6‐0 silk suture and punctured twice through‐and‐through with a 21‐gauge needle. The abdominal wall and incision were then closed with 6‐0 silk suture. Sham‐operated animals underwent laparotomy without ligation or puncture of the cecum. For sample collection, 24 h after the procedure, animals were euthanized with CO_2_ inhalation, and peritoneal lavages were performed with 4 mL of sterile saline and then blood was collected from the thoracic aorta. The animal experimental protocols were approved by the Review Committee of Zhejiang University School of Medicine and were in compliance with institutional guidelines.

### Enzyme‐Linked Immunosorbent Assay (ELISA)

Serum IL‐6 and CXCL1 levels were determined in duplicate in 96‐well half‐area plates using a standard plate reader. The assays were performed according to the manufacturer's instructions with appropriate dilutions.

### Tissue Histological Analyses

At 24 h after CLP, the mice were anesthetized and euthanized. The lung and kidney tissues were fixed in 4% paraformaldehyde for 24 h and then sectioned serially. A 4‐point scale (0 denoted normal lungs; 1, mild, less than 25% lung involvement; 2, moderate, 25–50% lung involvement; 3, severe, 50–75% lung involvement; and 4, very severe, >75% lung involvement) was used to evaluate lung damage based on alveolar congestion, capillary congestion, leukocyte or neutrophil infiltration, and thickness of the alveolar wall. Using an image analyzing system, leukocyte infiltration in the lung was estimated by quantitative morphometric analysis. Kidney damage was based on necrosis characterized by loss of architecture, vacuolization, and increased eosinophilia. A scale of 0 to 4 (0 denoted normal liver; 1, mild; 2, moderate; 3, severe; and 4, total necrotic destruction of the kidney) was used to evaluate kidney damage.

### Statistics Analysis

Data are presented as mean ± standard deviation (SD). For intergroup comparisons, independent‐sample *t*‐tests or paired *t*‐tests were applied to normally distributed variables, while Wilcoxon rank‐sum tests were used for non‐normally distributed variables. For multi‐group comparisons, one‐way ANOVA with Tukey–Kramer post hoc testing was performed for datasets exhibiting normal distribution and homogeneity of variance. Correlations were analyzed using Pearson's coefficient (*r*). The R package survminer was utilized to determine cutoff values for distinguishing moCD38 high and low expression subgroups. Survival differences (Kaplan–Meier curves) were evaluated via the log‐rank (Mantel–Cox) test. Univariate and multivariate Cox regression models assessed the association between moCD38 and 28‐day mortality. The DeLong method was employed for area under the curve (AUC) comparisons. Statistical significance was defined as a two‐tailed *p* < 0.05, with annotations as follows: ns (not significant, *p* > 0.05), ^*^ *p* < 0.05, ^**^ *p* < 0.01, ^***^ *p* < 0.001, ^****^ *p* < 0.0001. Analysis were conducted using SPSS (v 17.0), GraphPad Prism 8, and R v 4.0.5.

## Conflict of Interest

The authors declare no conflict of interest.

## Author Contributions

S.J., W.Y., and W.Y. conceived the idea and provided guidance. N.H., L.K., and L.F. prepared the samples and conducted the experiment. Y.B., Y.Z., H.Y., H.L., P.D., Y.N., and P.Y. discussed and analyzed the data. L.L., Y.X., L.Z., X.W., R.W., X.L., Q.Z. collected blood samples from patients and their clinical information. Q.J. contribute to the CyTOF experiments. N.H. wrote the manuscript with assistance from S.J., W.Y., and W.Y. The authors were all involved in interpreting and constructing the figures. N.H., L.K., and L.F. contributed equally to this work.

## Supporting information



Supporting Information

## Data Availability

The data that support the findings of this study are available from the corresponding author upon reasonable request.
