# Supplementary material for: Identification of CD38high Monocyte as a Candidate Diagnostic Biomarker and Therapeutic Target for Sepsis
Source: Adv Sci (Weinh). 2025 Mar 27;12(23):2500457. doi: 10.1002/advs.202500457 (PMC12199442; doi:10.1002/advs.202500457)
Supplement: Supplementary file 1 — Supporting Information [file ADVS-12-2500457-s001.docx]

**Table S1:** Baseline Characteristics of validation cohort1 Patients for CyTOF

| Characteristic | HC | Sepsis | Mild | Surgery | Recovery |
| --- | --- | --- | --- | --- | --- |
|  |  |  |  |  |  |
| Total | 20 | 35 | 12 | 20 | 11 |
| Male | 12 | 22 | 7 | 13 | 6 |
| Female | 8 | 13 | 5 | 7 | 5 |
| Age | 67(54-74) | 68(62-79) | 56(37-88) | 57(42-65) | 66(36-77) |
| WBC, 10E9/L, median (IQR） |  | 9.4(6.2-17.7) | 12.5(6.7-18.3) | 9.8(8.3-10.6) | 10.6(7.4-14.3) |
| CRP, mg/L, median (IQR） |  | 166.9(135.9-245.4) | 114(67.5-160.5)^**^ | 41.1(28.1-65.6)^***^ | 67.7(16.5-107.6)^***^ |
| PCT, ng/ml, median (IQR） |  | 17.9(9.2-26.6) | 1.3(0.3-2.3)^***^ | 0.7(0.4-2.5)^***^ | 0.4(0.2-2.9)^****^ |
| SCR, μmol/L, median (IQR） |  | 124.0(72.0-176.0) | 71.4(45.6-97.2)^**^ | 99.5(61.0-128.0)^*^ | 66.5(54.0-70.0)^**^ |
| ALT, U/L, median (IQR） |  | 31.8(15.0-56.6) | 28.3(12.5-44.1) | 16.0(11.5-30.5)^**^ | 30.0(12.0-48.0) |
| AST, U/L, median (IQR） |  | 40.9(23.2-58.5) | 23.9(12.6-35.2)^**^ | 38.8(21.2-65.8) | 25.5(12-33)^*^ |
| Bilirubin, μmol/L, median (IQR） |  | 34.2(19.1-56.3) | 13.1(5.3-20.9)^***^ | 16.4(8.9-22.8)^**^ | 15.3(7.2-22.6)^**^ |
| IL-6, pg/ml, median (IQR) |  | 1827.8(327.4-3149.8 | 814.6(451.2-1178)^***^ | 1539.2(647.4-2428.8) | 849.2(283.1-1482.5)^**^ |
| TNF-a, pg/ml, median (IQR) |  | 5.6(1.8-9.4) | 2.6(0.9-4.1)^**^ | 6.1(1.2-11.0) | 2.3(0.8-3.8)^***^ |
| Blood Glucose mmol/L(IQR) |  | 10.1(4.8-15.4) | 5.2(2.1-8.3)^***^ | 9.8(3.9-15.7)^*^ | 5.8(2.5-9.1)^***^ |
| SOFA, median (IQR) |  | 9.5(6.0-12.0) |  | 6.0(3.0-9.0)^**^ |  |
| APACHE II, median (IQR) |  | 20.0(11.0-29.0) |  | 14.0(11.0-17.0)^**^ |  |
| underlying disease No. (%) |  |  |  |  |  |
|  |  |  |  |  |  |
| diabetes |  | 9.0(25.7%) |  | 0.0(0.0%) | 5.0(45.5%) |
| hypertension |  | 12.0(34.3%) |  | 10.0(50.0%) | 5.0(45.5%) |
| Cardiovascular disease |  | 5.0(14.3%) |  | 2.0(10.0%) | 2.0(18.2%) |
| chronic pulmonary disease |  | 3(8.6%) |  | 0(0.0%) | 1(9.1%) |
| Source of infection No.(%) |  |  |  |  |  |
| respiratory tract infection |  | 12(34.3%) |  |  | 6(54.5%) |
| Abdominal infection |  | 18(51.4%) |  |  | 3(27.3%) |
| urinary tract infection |  | 5(22.9%) |  |  | 2(18.2%) |
| Pathogenic microorganisms No. (%) |  |  |  |  |  |
| Bacteria |  |  |  |  |  |
| >1 positive culture |  | 9(25.7%) |  |  |  |
| 1 postive culture |  | 16(64.0%) |  |  |  |
| *Klebsiella pneumoniae* |  | 3(8.6%) |  |  |  |
| *Acinetobacter baumannii* |  | 2(5.7%) |  |  |  |
| *Escherichia coli* |  | 1(2.9%) |  |  |  |
| *Pseudomonas aeruginosa* |  | 2(5.7%) |  |  |  |
| *Enterobacteriaceae* |  | 2(5.7%) |  |  |  |
| *Staphylococcus aureus* |  | 2(5.7%) |  |  |  |
| Other bacteria |  | 4(11.4%) |  |  |  |
| Other pathogens |  |  |  |  |  |
| Fungus |  | 4(16.0%) |  |  |  |
| Miscellaneous |  | 6(24.0%) |  |  |  |

Categorical variables are expressed as n (%), and continuous variables were expressed as median (interquartile range). Comparisons between Sepsis patients and other group patients were performed with chi-square or Fisher exact tests for qualitative variables and Mann Whitney U for quantitative variables. * *P* < 0.05, ** *P* < 0.01, *** *P* < 0.001, **** *P* < 0.0001. *WBC*, white blood cell; *CRP*, C-reactive protein; *PCT*, procalcitonin; SCR, serum creatinine; *ALT*, glutamic pyruvic transaminase; *AST*, glutamic oxaloacetic transaminase; IL-6, interleukin-6; TNF-α, tumor necrosis factor α; *SOFA*, sequential organ failure assessment; *APACHE II*, acute physiology and chronic health evaluation II.

**Table S2:** Baseline Characteristics of validation cohort 2 Patients for FC

| Characteristic | Sepsis | BS subgroup | OS subgroup | Surgery | Mild |
| --- | --- | --- | --- | --- | --- |
|  |  |  |  |  |  |
| Total | 102 | 71 | 31 | 98 | 53 |
| Male | 70 | 49 | 21 | 47 | 29 |
| Female | 32 | 22 | 10 | 51 | 24 |
| Age | 66(56-75) | 61(56-74) | 64(61-75) | 63(51-73) | 66(50-76) |
| WBC, 10E9/L, median (IQR） | 12.8(2.1-22.5) | 12.5(6.3-19.3) | 10.1(4.7-17.2)^*^ | 9.9(7.7-11.8)^**^ | 8.1(3.1-16.8)^**^ |
| CRP, mg/L, median (IQR） | 150.0(58.9-241.4) | 149.2(58.9-241.4) | 128.4(28.3-171.7)^**^ | 45.6(11.1-69.8)^***^ | 76.6(19.9-150.8)^***^ |
| PCT, ng/ml, median (IQR） | 12.3(0.9-82.3) | 11.3(1.20-82.3) | 10.1(0.9-67.3) | 1.9(0.1-3.9)^***^ | 0.45(0.1-1.2)^****^ |
| SCR, μmol/L, median (IQR） | 121.0(65.0-181.0) | 101.0(65.0-181.0) | 131.0(51.0-171.0) | 91.0(51.0-110.5)^**^ | 68.6(43.2-109.8)^***^ |
| ALT, U/L, median (IQR） | 39.0(8.1-293.9) | 32.0(8.0-68.0) | 27.0(12.0-59.2)^*^ | 28.6(6.0-56.8)^*^ | 29.2(9.8-59.1)^*^ |
| AST, U/L, median (IQR） | 69.0(9.0-102.7) | 64.0(17.0-89.1) | 78.2(29.1-102.3)^**^ | 48.1(21.6-69.5)^**^ | 35.8(17.6-62.9)^***^ |
| Blood Glucose mmol/L(IQR) | 9.8(4.1-15.5) | 10.0（3.9-16.1） | 9.8(4.2-15.4)^**^ | 9.6(3.6-15.6)^*^ | 6.9(2.5-11.9)^***^ |
| SOFA, median (IQR) | 10.0(5.0-16.0) | 9.0(5.0-14.0) | 10.0(6.0-15.0)^*^ | 6.0(4.0-8.0)^**^ |  |
| APACHE II, median (IQR) | 24.3(12.3-32.7) | 17.2(12.3-32.7) | 19.2(14.1-27.9)^*^ | 13.0(5.0-20.0)^**^ |  |
| underlying disease No. (%) |  | | | | |
|  |  |  |  |  |  |
| diabetes | 18(17.7%) | 12(16.9%) | 6(19.3%) |  |  |
| hypertension | 42(41.2%) | 25(35.2%) | 17(54.8%) |  |  |
| Cardiovascular disease | 27(26.5%) | 18(25.4%) | 9(29.0%) |  |  |
| chronic pulmonary disease | 8(7.8%) | 6(8.45%) | 2(6.5%) |  |  |
| Source of infection No. (%) |  |  |  |  |  |
| bloodstream infection | 12(11.8%) | 7(9.9%) | 5(16.1%) |  |  |
| respiratory tract infection | 29(28.4%) | 19(26.8%) | 10(32.3%) |  |  |
| Abdominal infection | 31(30.4%) | 24 (33.8%) | 7(22.6%) |  |  |
| urinary tract infection | 10(9.8%) | 8(11.3%) | 2(6.5%) |  |  |
| other infection | 20(19.6%) | 13(18.3%) | 7(22.6%) |  |  |
| Pathogenic microorganisms No. (%) |  |  |  |  |  |
| Bacteria |  |  |  |  |  |
| >1 positive culture | 21(20.0%) |  |  |  |  |
| 1 positive culture | 50(49.0%) |  |  |  |  |
| *Klebsiella pneumoniae* | 13(12.7%) |  |  |  |  |
| *Acinetobacter baumannii* | 4(3.9%) |  |  |  |  |
| *Escherichia coli* | 6(5.9%) |  |  |  |  |
| *Enterobacteriaceae* | 9(8.8%) |  |  |  |  |
| *Mycobacterium tuberculosis* | 4(3.9%) |  |  |  |  |
| *Staphylococcus aureus* | 5(4.9%) |  |  |  |  |
| Other bacteria | 9(8.8%) |  |  |  |  |
| Other pathogens |  |  |  |  |  |
| Fungus | 21(20.6%) |  |  |  |  |
| Miscellaneous | 10(9.8%) |  |  |  |  |

Categorical variables are expressed as n (%), and continuous variables were expressed as median (interquartile range). Comparisons between BS subgroup Sepsis patients and other group patients were performed with chi-square or Fisher exact tests for qualitative variables and Mann Whitney U for quantitative variables. * *P* < 0.05, ** *P* < 0.01, *** *P* < 0.001, **** *P* < 0.0001. *WBC*, white blood cell; *CRP*, C-reactive protein; *PCT*, procalcitonin; *SCR*, serum creatinine; *ALT*, glutamic pyruvic transaminase; *AST*, glutamic oxaloacetic transaminase; *IL-6*, interleukin-6; *TNF-α*, tumor necrosis factor α; *SOFA*, sequential organ failure assessment; *APACHE II*, acute physiology and chronic health evaluation II; BS, bacterial sepsis; OS, other pathogen sepsis.

**Supporting information Figures**


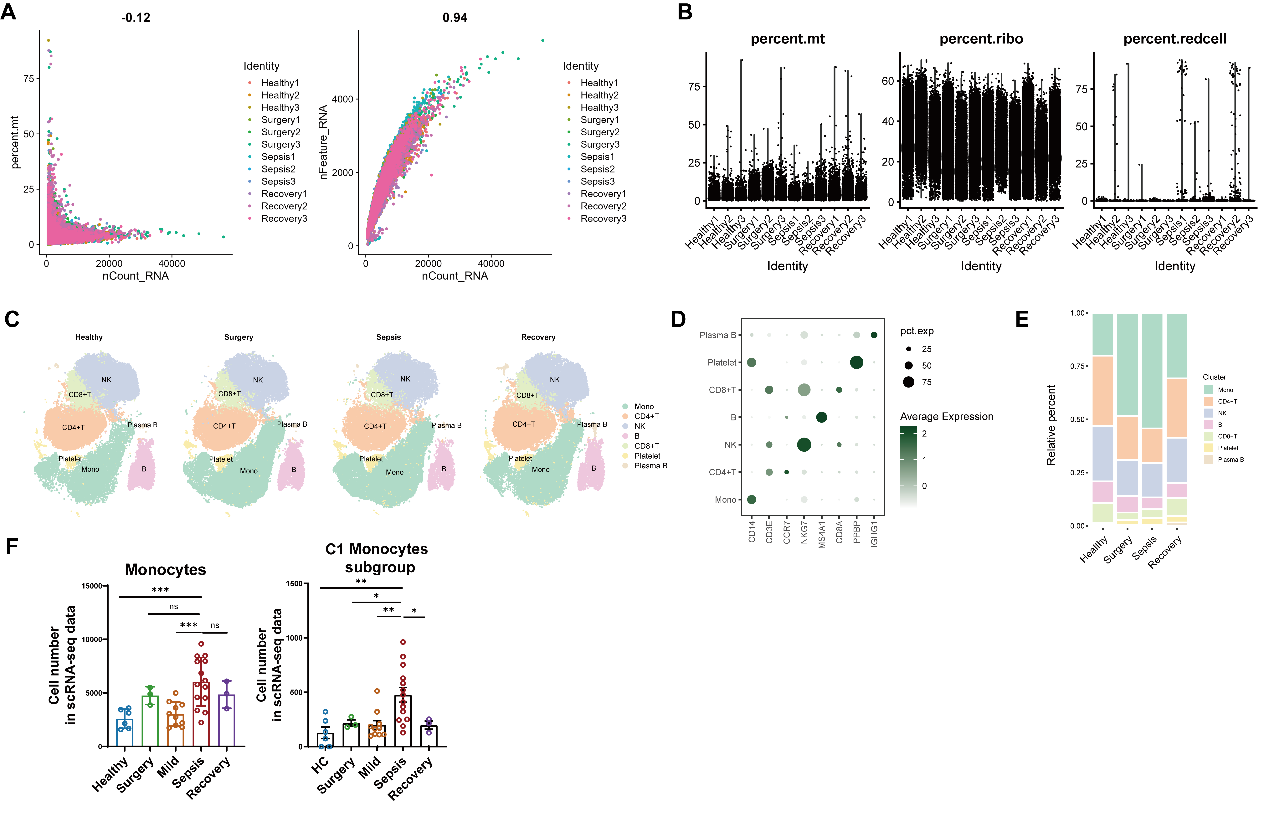


**Figure S1: Identification of cell types in sepsis by scRNA-seq, Related to Figure 1**

A) Scatter plots showing the quality control metrics used in the scRNA-seq analysis. The percentage of reads that map to the mitochondrial genome was shown in (left), and the number of unique genes detected in each cell was shown in (right). B) Violin plots of various quality control metrics for the full scRNA-seq dataset generated in this study. C) t-SNE plot for cellular heterogeneity with seven distinct cell clusters identified in each group. General identity of each cell cluster is annotated to the right. D) Average cell percentage of each cell type derived from four groups. E) Dot plot showing the average expression of representative marker genes for each cell type (Healthy, n = 6; Surgery, n = 3; Mild, n = 10; Sepsis, n = 14; Recovery, n = 3). F) The cell numbers of monocytes and the C1 monocyte subpopulation from different groups in the single-cell transcriptome data (Healthy, n = 6; Surgery, n = 3; Mild, n = 10; Sepsis, n = 14; Recovery, n = 3). Error bars represent mean ± SD.* *P* < 0.05; ** *P* < 0.01; ns, no significant difference (*P* > 0.05) (One-way ANOVA and Tukey post hoc tests for F).


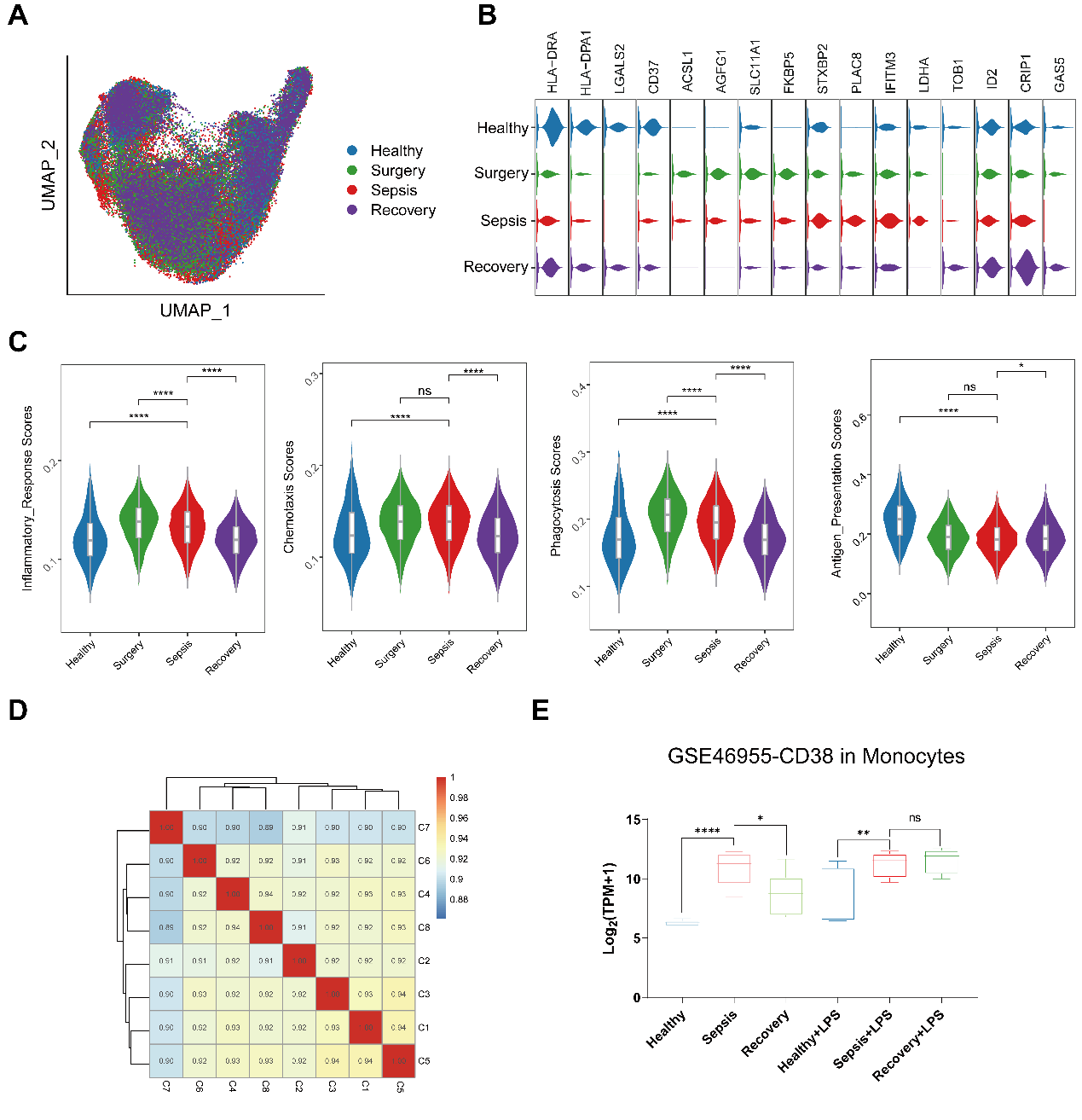


**Figure S2: scRNA-seq analysis revealed heterogeneity of monocytes in sepsis, Related to Figure 1**

A) UMAP plot for integrated monocytes; each dot corresponds to a single cell, colored according to different groups. B) Violin plot showing the expression of top 4 marker genes (sorted by “avg_log2FC”) for different groups of monocytes. C) Violin plot of Inflammatory Response score, Chemotaxis score, Phagocytosis score, and Antigen presentation score in monocytes of different groups. (two sided Wilcoxon rank-sum test). * *P* < 0.05; **** *P* < 0.0001, ns, no significant difference (*P* > 0.05). D) Heatmap showing the Spearman’s correlation between subclusters of monocytes. E) The expression of *CD38* in monocytes from healthy donors, septic patients, and those who recovered from sepsis with or without LPS. Data sourced from the GEO database (GSE46955). (n = 6, 8, 8 respectively; mean ± SD). * *P* < 0.05; ** *P* < 0.01; *** *P* < 0.001; **** *P* < 0.0001; ns, no significant difference (*P* > 0.05) (One-way ANOVA and Tukey post hoc tests).


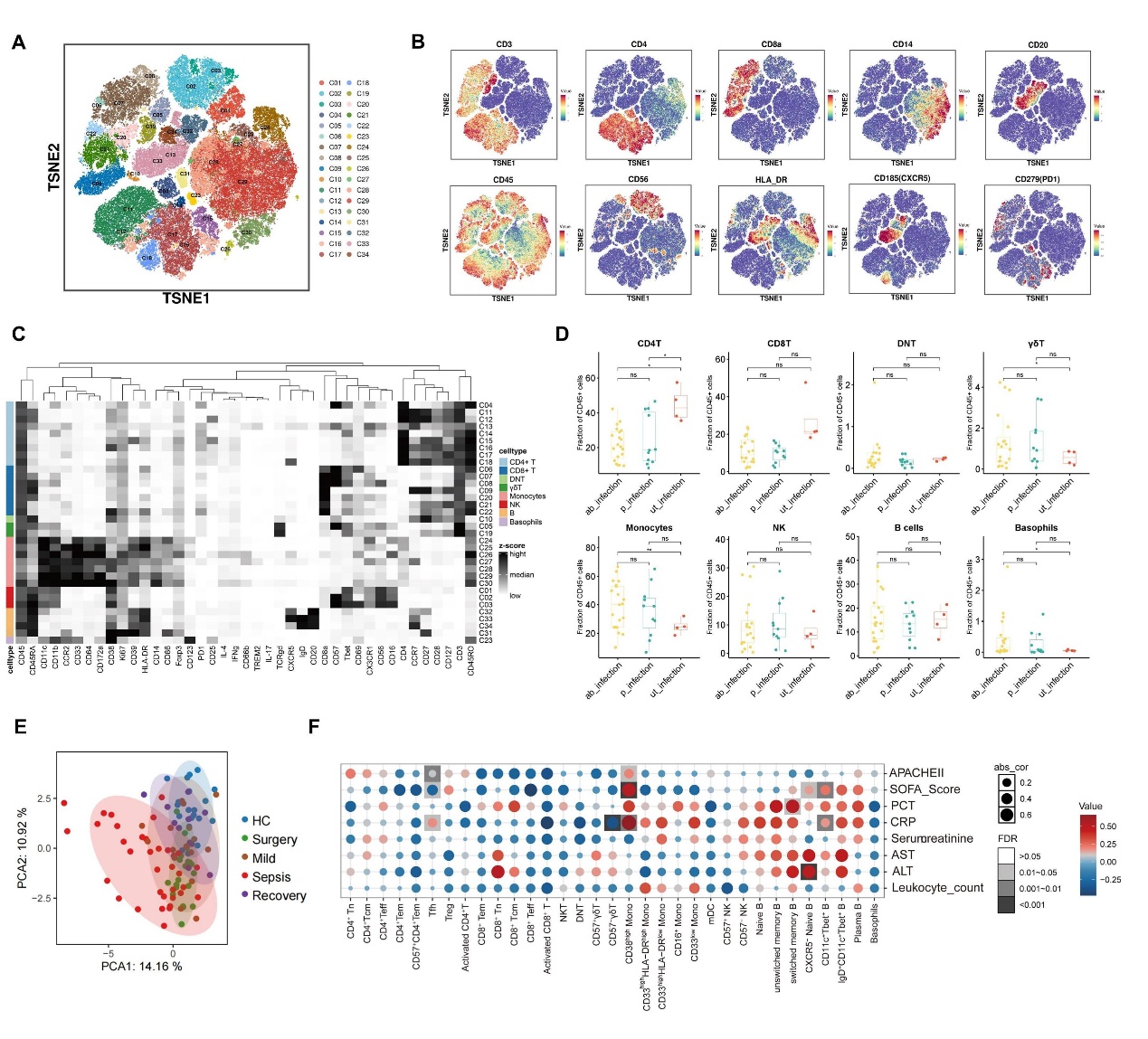


**Figure S3: Immune Cell Phenotyping in sepsis, Related to Figure 2**

A) t-SNE maps displaying PBMC analyzed with immune cell subsets in healthy controls, surgery patients, septic patients, and patients recovered from sepsis diseases. We analyzed 34 clusters within immune cells. B) t-SNE plot showing markers analyzed with immune molecules. C) Heatmap of immune cells subsets. Clustering analysis for markers distinguishing CD4^+^ T cells, CD8^+^ T cells, double negative T cells，γδT cells, monocytes, NK cells, B cells, and basophils. D) Comparisons of the percentages of major immune cell subsets across different infection sources of sepsis, including abdominal infection (ab_infection), pulmonary infection (p_infection) and urinary tract infection (ut_infection). Statistical significance was determined by a two-sided, unpaired Wilcoxon rank-sum test. * *P* < 0.05; ** *P* < 0.01; *** *P* < 0.001; **** *P* < 0.0001; ns, no significant difference (*P* > 0.05). All boxplots show median, first and third quartiles; whiskers 1.5x interquartile range. E) PCA plot showing the distribution of different groups in CyTOF. F) Covariate analysis of cell abundance assayed by CyTOF and clinical, and experimental variables for sepsis cases (BH adjusted ANOVA test for significance).


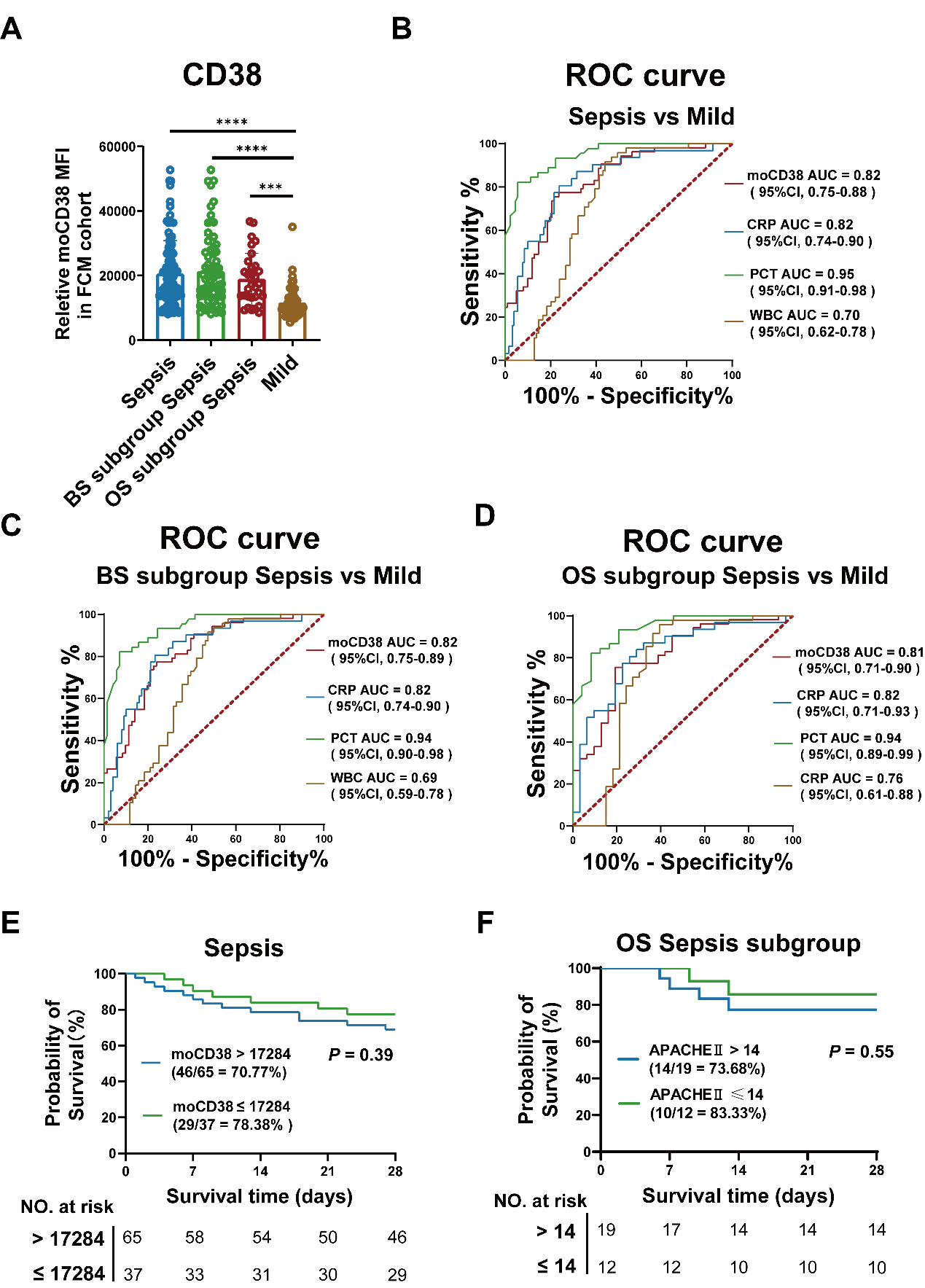


**Figure S4: Sepsis-specific CD38^high^ monocytes are detectable by flow cytometry and discriminate sepsis patients from non-infected patients, Related to Figure 3**

A) Mean fluorescence intensity of CD38 in monocytes from whole blood of sepsis patients (n = 102), patients with bacterial sepsis (BS) (n = 71), patients with other pathogens sepsis (OS) (n = 31), and patients with mild infection (n = 51). Statistical significance was determined by a One-way ANOVA and Tukey post hoc tests. *** *P* < 0.001; **** *P* < 0.0001. Error bars represent mean ± SD. B) Receiver operating characteristic curve showing the performance of the expression levels of CD38 and clinical indexes in monocytes in distinguishing sepsis patients (n = 102) from Mild infectious patients (n = 53). C) Receiver operating characteristic curve showing the performance of the expression levels of CD38 and clinical indexes in monocytes in distinguishing bacterial sepsis patients (n = 71) from Mild infectious patients (n = 53). D) Receiver operating characteristic curve showing the performance of the expression levels of CD38 and clinical indexes in monocytes in distinguishing other pathogens sepsis patients (n = 31) from Mild infectious patients (n = 53). E) Kaplan-Meier survival curves of 102 patients with sepsis based on the expression levels of CD38 in monocytes cutoff value (17284) on day of ICU admission. F) Kaplan-Meier survival curves of 31 patients with sepsis based on APACHE Ⅱ cutoff value (14) on day of ICU admission.


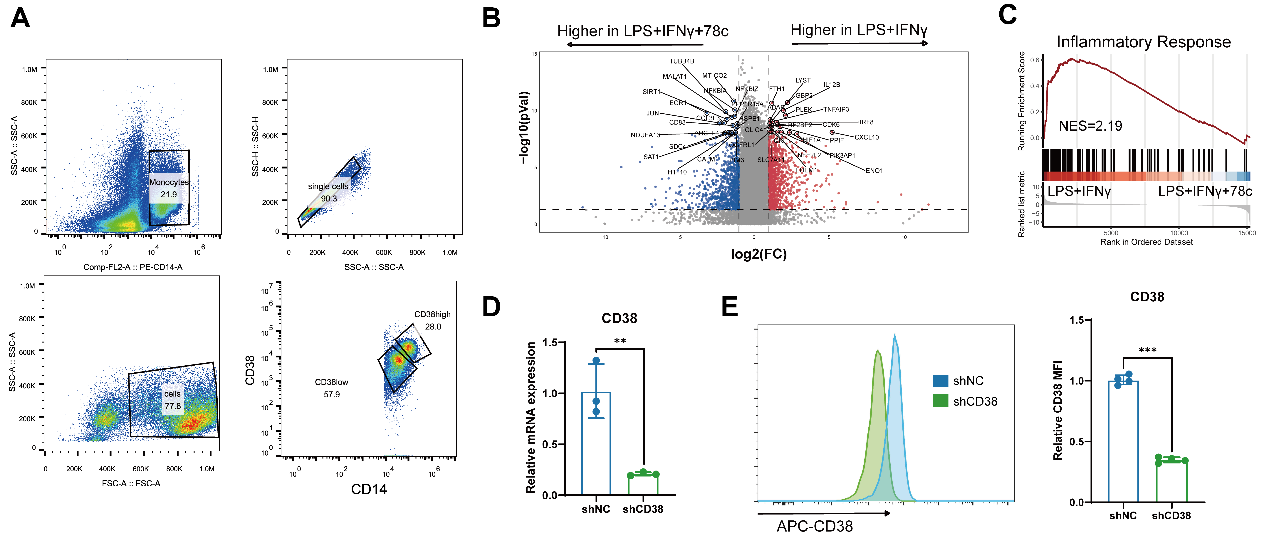


**Figure S5: Inhibition of CD38 changed the inflammatory response of monocytes, Related to Figure 4**

A) Peripheral blood-derived monocytes were analyzed by flow cytometry (FACS) in terms of the expression of CD14 and CD38, and CD38 expression levels as shown in this representative gating strategy. B) Volcano plot representation of significantly up- and downregulated genes. C) Gene Set Enrichment Analysis (GSEA) plot of enrichment in “Inflammatory Response” signature. D) qPCR analysis of *CD38* expression in cultured monocytes THP-1 with or without *CD38* knockdown normalized to *ACTB* (n = 3 per group). E) CD38 expression in cultured monocytes THP-1 with or without *CD38* knockdown measured by Flow cytometry (n = 4 per group). All data are representative of at least two independent experiments performed in triplicate. Error bars represent mean ± SD. ** *P* < 0.01; *** *P* < 0.001. Two-tailed unpaired Student’s t-test was used in D, E.


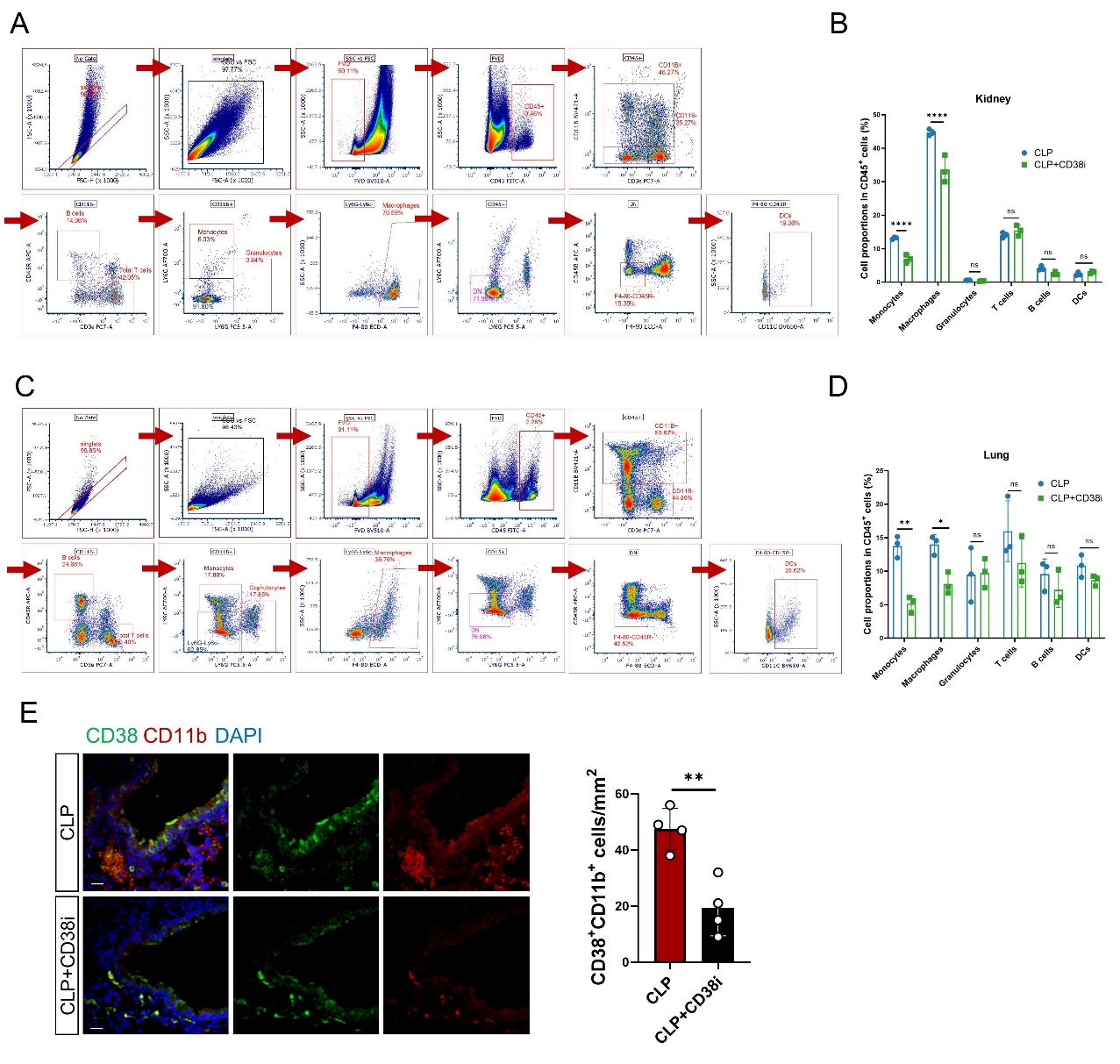


**Figure S6: Inhibition of CD38 altered the CD38^high^ monocytes and macrophages in the kidney and lung *in vivo*, Related to Figure 4**

A) The gating strategy applied on fluorescent flow cytometry data of immune cells from the kidneys. B) Comparisons of the differences in immune cell composition in the kidney tissues upon CD38 inhibition (n = 3 per group). C) The gating strategy applied on fluorescent flow cytometry data of immune cells from the Lungs. D) Comparisons of the differences in immune cell composition in the lung tissues upon CD38 inhibition (n = 3 per group). E) Immunofluorescence staining of lung tissue for CD38 (green) and CD11b (red) (n = 4 per group; scale bar = 50 μM). Error bars represent mean ± SD. ** *P* < 0.01; *** *P* < 0.001. Two-tailed unpaired Student’s t-test was used in B, D, E.


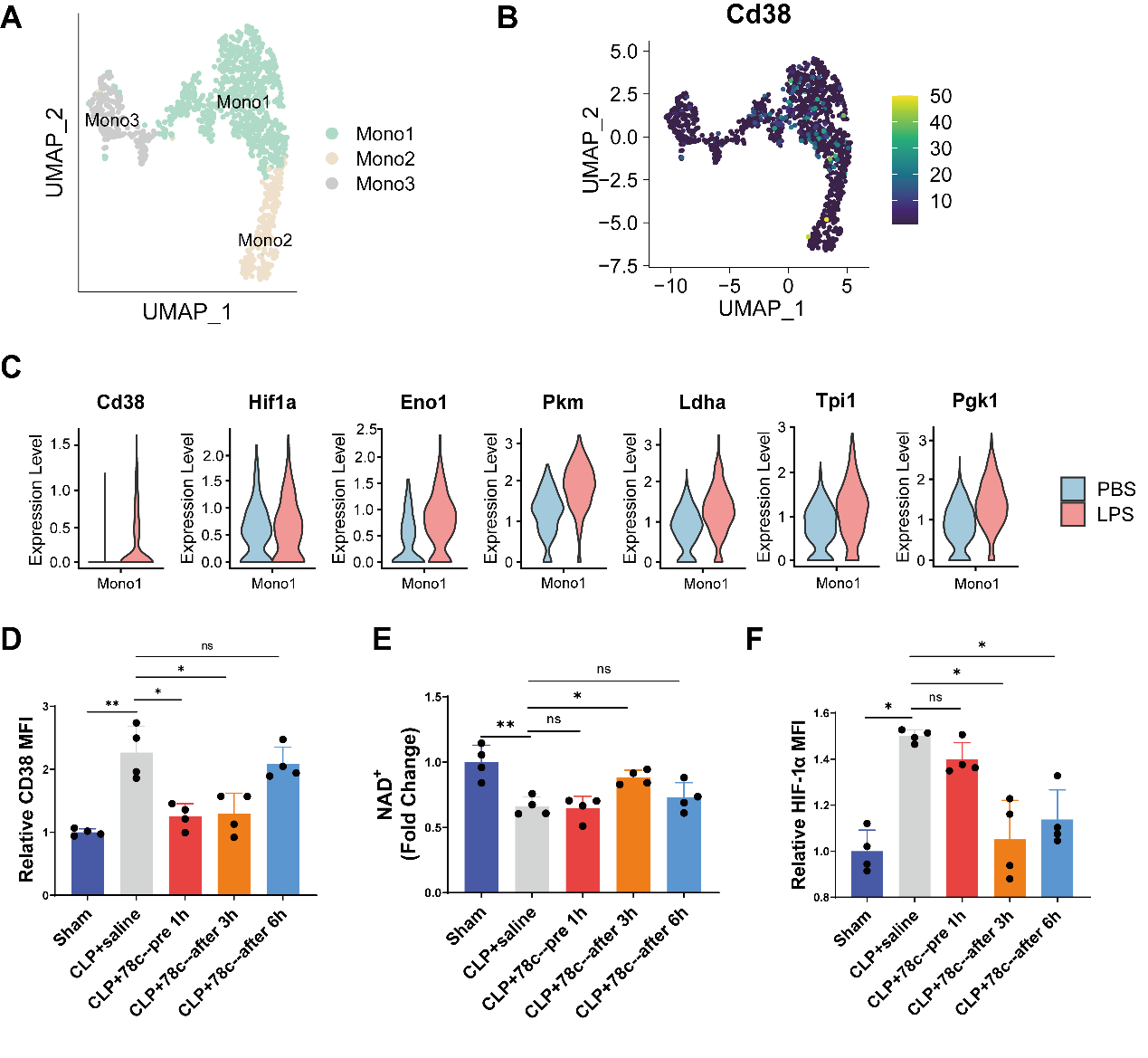


**Figure S7:** **CD38 blocking treatment reduce the glycolytic activity of sepsis model, Related to Figure 6**

A) UMAP plot of cultured murine monocytes derived from bone marrow. B) Normalized expression of CD38 is color coded and projected on the UMAP plot. C) Violin plot showing expression level of selected genes of two groups in Mono1. D) Expression levels of CD38 were assessed by flow cytometry on mouse CD14^+^ monocytes with different treatments (n = 4 per group). E) Measurement of NAD^+^ levels of mouse monocytes from sepsis model (n = 4 per group). F) Expression levels of HIF-1a were assessed by flow cytometry on mouse CD14^+^ monocytes with different treatments (n = 4 per group). All data are representative of at least two independent experiments performed in triplicate. Error bars represent mean ± SD.* *P* < 0.05; ** *P* < 0.01; *** *P* < 0.001; **** *P* < 0.0001; ns, no significant difference (*P* > 0.05). One-way ANOVA and Tukey post hoc tests for D, E, F.


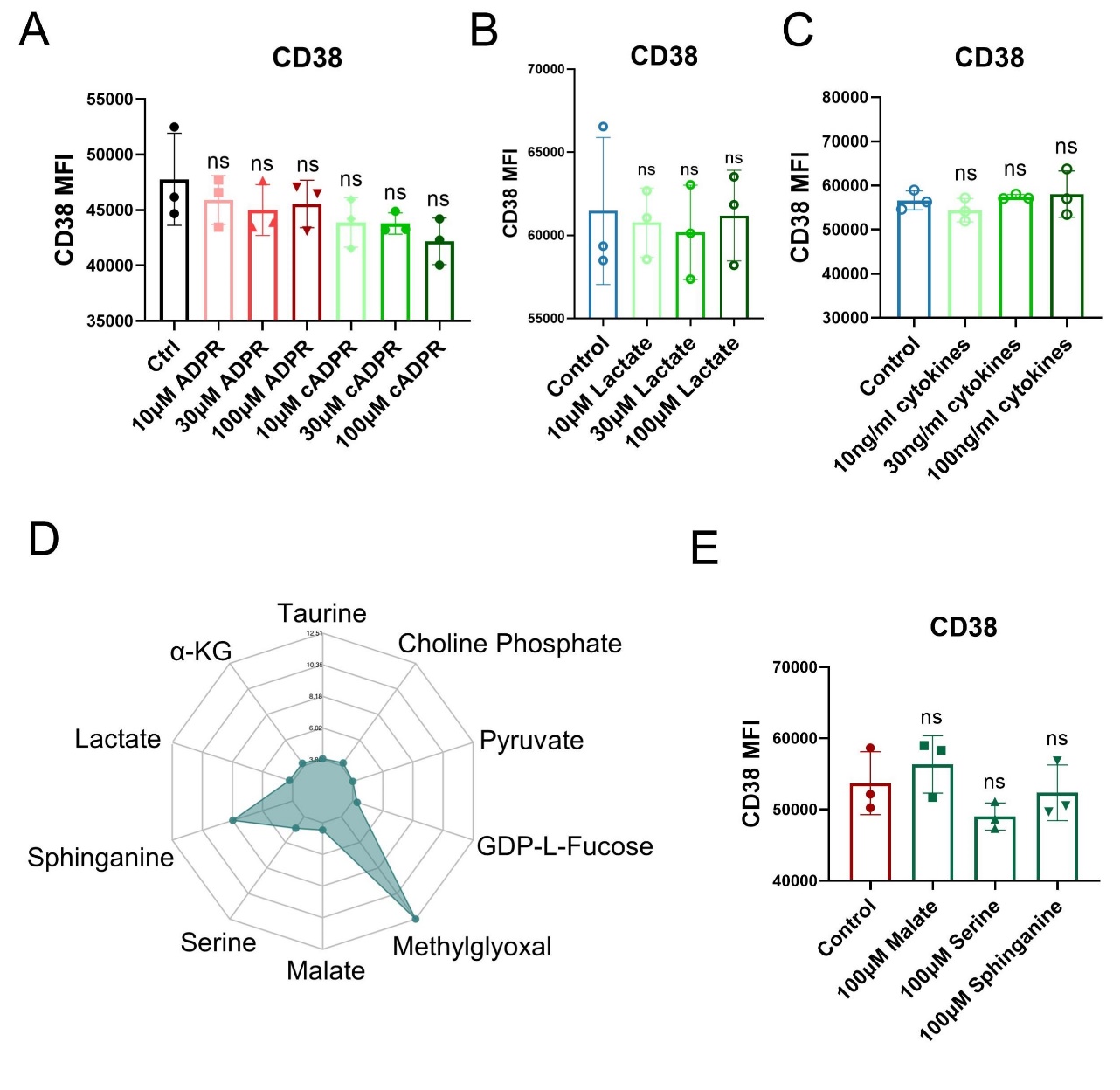


**Figure S8: Screening metabolites and cytokines for their impact on the expression of CD38 in monocytes, Related to Figure 7**

A) Investigation of the effects of cADPR and cADPR treatment on the expression of CD38 in monocytes (n = 3 per group). B) Investigation of the effects of lactate treatment on the expression of CD38 in monocytes (n = 3 per group). C) Investigation of the effects of cytokines (IL6 + TNFa + IL1β) treatment on the expression of CD38 in monocytes (n = 3 per group). D) Radar plot showing highly expressed metabolites in the conditioned media of CD38^high^ monocytes. E) Investigation of the effects of several metabolites treatment on the expression of CD38 in monocytes (n = 3 per group). All data are representative of at least two independent experiments performed in triplicate. Error bars represent mean ± SD. ns, no significant difference (*P* > 0.05). One-way ANOVA and Tukey post hoc tests for A, B, C, E.
